# A comprehensive overview of liquid biopsy applications in pediatric solid tumors

**DOI:** 10.1038/s41698-024-00657-z

**Published:** 2024-08-03

**Authors:** Ferdinand W. Janssen, Nathalie S. M. Lak, Claudia Y. Janda, Lennart A. Kester, Michael T. Meister, Johannes H. M. Merks, Marry M. van den Heuvel-Eibrink, Max M. van Noesel, Jozsef Zsiros, Godelieve A. M. Tytgat, Leendert H. J. Looijenga

**Affiliations:** 1 Princess Máxima Center, Utrecht, the Netherlands; 2 Oncode Institute, Utrecht, the Netherlands; 3 Division of Imaging and Oncology, University Medical Center Utrecht, University of Utrecht, Utrecht, the Netherlands; 4 Wilhelmina Children’s Hospital-Division of CHILDHEALTH, University Medical Center Utrech, University of Utrecht, Utrecht, the Netherlands; 5 Department of Genetics, University Medical Center Utrecht, University of Utrecht, Utrecht, the Netherlands; 6 Department of Pathology, University Medical Center Utrecht, University of Utrecht, Utrecht, the Netherlands

**Keywords:** Diagnostic markers, Predictive markers, Prognostic markers, Paediatric cancer, Paediatric cancer

## Abstract

Liquid biopsies are emerging as an alternative source for pediatric cancer biomarkers with potential applications during all stages of patient care, from diagnosis to long-term follow-up. While developments within this field are reported, these mainly focus on dedicated items such as a specific liquid biopsy matrix, analyte, and/or single tumor type. To the best of our knowledge, a comprehensive overview is lacking. Here, we review the current state of liquid biopsy research for the most common non-central nervous system pediatric solid tumors. These include neuroblastoma, renal tumors, germ cell tumors, osteosarcoma, Ewing sarcoma, rhabdomyosarcoma and other soft tissue sarcomas, and liver tumors. Within this selection, we discuss the most important or recent studies involving liquid biopsy-based biomarkers, anticipated clinical applications, and the current challenges for success. Furthermore, we provide an overview of liquid biopsy-based biomarker publication output for each tumor type based on a comprehensive literature search between 1989 and 2023. Per study identified, we list the relevant liquid biopsy-based biomarkers, matrices (e.g., peripheral blood, bone marrow, or cerebrospinal fluid), analytes (e.g., circulating cell-free and tumor DNA, microRNAs, and circulating tumor cells), methods (e.g., digital droplet PCR and next-generation sequencing), the involved pediatric patient cohort, and proposed applications. As such, we identified 344 unique publications. Taken together, while the liquid biopsy field in pediatric oncology is still behind adult oncology, potentially relevant publications have increased over the last decade. Importantly, steps towards clinical implementation are rapidly gaining ground, notably through validation of liquid biopsy-based biomarkers in pediatric clinical trials.

## Introduction

### Pediatric cancer

Cancer is characterized by uncontrolled malignant cell proliferation following accumulated (epi)genetic aberrations^1^. In children between 0 and 19 years, globally 289,500 new cases were diagnosed in 2020^2^. Fortunately, increased insight in cancer biology and tumorigenesis together with international clinical trials enabled advances in treatment and supportive care and led to improved survival rates^3,4^. Indeed, in the US, the 5-year overall survival (OS) rate for children improved from 58% during the 1970s to 85% during 2012 through 2018^5^. Nonetheless, and despite being a rare disease, cancer remains the leading cause of disease-related death in children over one year of age in high-income countries worldwide^6^. Moreover, despite of the rising cure rates, significant long-term side effects exist, for which reduction of therapy burden is also an important target^7^.

Pediatric and adult cancers are distinctly different regarding cause, occurrence, (epi)genetic properties, and outcome^8^. The most common pediatric cancer types include leukemias, central nervous system tumors, lymphomas, neuroblastoma, sarcomas (bone and soft tissue tumors), renal tumors, and germ cell tumors^5^. In contrast, breast, prostate, lung, and colorectal cancers are the most common types among adults^5^. Pediatric cancers generally have a low mutational burden with few recurrent hotspots and are characterized by epigenetic dysregulation and chromosomal structural variations (SVs), including copy number alterations (CNAs), and translocations^8–11^. Adult cancers are characterized by a high number of somatic mutations and insertion/deletion errors^8,10^. It is suggested that the mutational landscape of adult cancers is a result of the accumulated effects of aging and long-term exposure to physical, chemical, and biological environmental factors^6,12–14^. In contrast, pediatric cancer origins are thought to primarily involve accumulations of (epi)genetic aberrations during embryonal development, and inheritance of pathogenic germline variants^10,11,15,16^.

Common cancer treatments include surgery, systemic treatment (chemotherapy, targeted therapy, hormonal therapy, and immunotherapy), and radiation therapy, depending on cancer type and stage^17^. Treatment-related side-effects such as immunosuppression and infection, nephrotoxicity, cardiotoxicity, ototoxicity, decreased fertility, pain, endocrine insufficiency, nausea and vomiting, and psychosocial effects have a significant impact on children and their quality of life^18,19^. Therefore, treatment should be optimally tailored for every child to maximize the likelihood of cure while minimizing short and long-term side effects. Currently, tumor biopsies are still the golden standard for disease diagnosis, tumor biology analysis, and therapeutic decision-making. However, these biopsies are invasive, can be difficult to obtain, and do not comprise full tumor heterogeneity^20^. This limits feasibility of tumor biopsies for serial sampling, monitoring of disease and treatment efficacy, and identification of treatment resistant clones^3^. Furthermore, patients often receive advanced imaging during diagnosis and disease monitoring, exposing them to ionizing radiation and/ or general anesthesia^21^. These imaging techniques are not sensitive enough to detect minimal residual disease and do not allow analysis of molecular changes during treatment^22^. Indeed, many children still relapse despite showing complete remission based on clinical imaging^3^. Relapse arises from drug-resistant minimal residual disease (MRD) cells throughout treatment at levels below morphological detection limits, and is the primary cause of pediatric cancer-related death^4^.

Taken together, pediatric cancers are clearly distinct cancers with unique diagnostic and therapeutic challenges, and there is an urgent need for alternative less-invasive biomarkers for diagnosis, disease monitoring, therapeutic decision-making, and early relapse detection^3,6^.

### Liquid biopsies

**Fig. 1:**
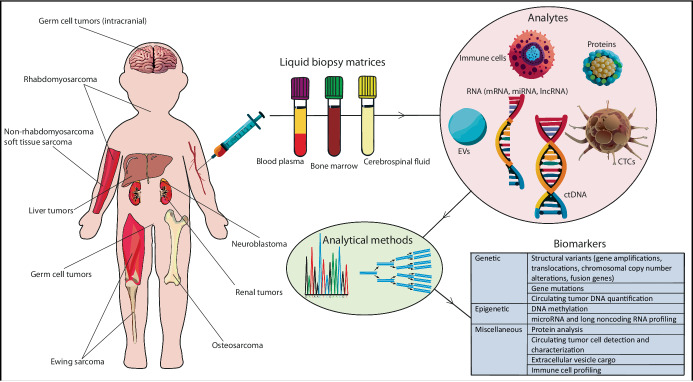
Graphical overview of liquid biopsy workflow for pediatric solid tumors. Frequent anatomical locations where pediatric solid tumors commonly present are indicated. Liquid biopsy matrices (e.g., peripheral blood, bone marrow, and CSF) are sampled and processed for isolation of analytes (e.g., ctDNA, CTCs, RNA (mRNA, miRNA, lncRNA), immune cells, and EVs) that are measured and characterized by specific methods (mostly PCR-based or sequencing-based) for biomarker analysis. The AI vector generator function from Adobe Illustrator was used for detailed visualization of this figure.

Malignant solid tumors are dynamic and continuously changing their microenvironment to enable growth and spread^23,24^. This includes shedding intact tumor cells and tumor-derived genetic material into the body fluids^24^. Sampling of these fluids, also known as a ‘liquid biopsy’, can be used for the detection and analysis of such tumor-derived material. Peripheral blood (plasma and serum) and cerebrospinal fluid (CSF) are the most established liquid biopsy fluids, with CSF being the most common for brain tumors^25,26^. Other body fluids such as bone marrow, urine, feces, saliva, sputum, pleural effusions, and ascites are also potentially useful liquid biopsies, depending on the tumor type^26–28^. Liquid biopsies provide access to different molecular analytes of which cell-free DNA (cfDNA)-derived circulating tumor DNA (ctDNA) is the most widely used^3,4,8,26,28–33^. In addition, RNA (cell-free RNA (cfRNA), messenger RNA (mRNA), microRNA (miRNA), long noncoding RNA (lncRNA)), circulating tumor cells (CTCs) including derived material, extracellular vesicle (EV) content, defined immune cell profiles, proteins, tumor-educated platelets, fibroblasts, and endothelial cells have also been described as analytes within liquid biopsies^33–35^ (Supplementary Tables 2–9). These analytes provide a source of potential biomarkers for cancer detection and monitoring^3^. A graphical overview of a liquid biopsy workflow is presented in Fig. 1.

Gradually, liquid biopsies are emerging as a more sensitive and potentially cheaper alternative to conventional tissue biopsies^32^. Clinical applications include (early) cancer detection, detection of therapeutic targets, real-time monitoring of therapeutic efficacy, monitoring tumor evolution and resistance mechanisms, early detection and prediction of relapse, and guidance of treatment decisions^9,33,36^. This offers the potential to monitor disease burden complementary or independently of radiologic or surgical findings. Also, while a tissue biopsy only provides information about the sampled region, a liquid biopsy captures the diverse tumor landscape, mitigating intra-tumor heterogeneity and temporal heterogeneity (differences among primary tumor and relapses)^3,8^. Finally, liquid biopsies are less-invasive than tissue biopsies and could avoid sedation, making them especially interesting for pediatric oncology^8,32^. Taken together, liquid biopsies can overcome several limitations associated with tissue biopsies.

**Fig. 2:**
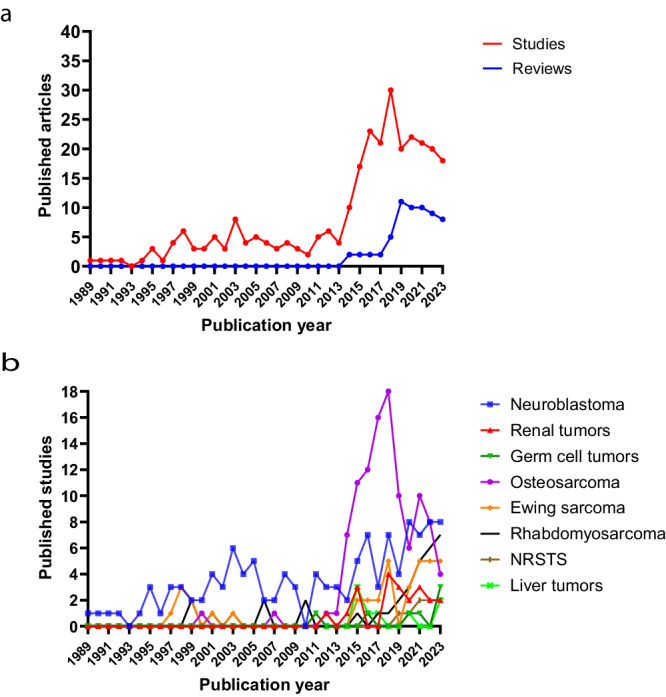
Historical overview of publications focusing on liquid biopsies in pediatric solid tumors. In (**a**), the number of unique articles (y-axis) is plotted over time (x-axis), for studies and review articles. All articles used for this plot are also included in Table 1. All articles in the ‘studies’ plot are also included in (**b**) and Supplementary Tables 2–9. All articles in the ‘reviews’ plot are also included in Supplementary Table 10. In (**b**), The number of published studies (y-axis) is plotted over time (x-axis), for each tumor type. Studies that included pediatric patients for more than one tumor type are indicated in each respective plot. All studies used for this plot are also included in (**a**), Table 1, and Supplementary Tables 2–9.

Liquid biopsies have already been implemented in adult oncology as Food and Drug Administration (FDA) approved screening and diagnostic assays, mostly based on the detection of specific recurring mutations using quantitative PCR (qPCR) and next-generation sequencing (NGS) approaches^37–40^. Examples of approved methods include CellSearch CTC detection, Cobas *EGFR* mutation test v2, Guardant 360 CDx, FoundationOne Liquid CDx, and Archer LIQUID*Plex*^40,41^. However, these specific panels cannot be translated to pediatric cancers due to the earlier discussed differences with adult cancer^8^. Indeed, at the time of publication, no liquid biopsy-based tests had been FDA-approved in pediatric oncology. Unique challenges such as sampling limitations and hence scarce input material (especially in young children), and readouts beyond mutational burden are provided^8^. Also, analytical method validation through large randomized controlled trials is required before clinical implementation^3,8^. Nonetheless, the field of liquid biopsies in pediatric oncology is rapidly expanding, and key findings for different tumor types are occasionally reviewed in the literature (Fig. 2a, Table 1, and Supplementary Table 10).

**Table 1:** Comprehensive overview of identified literature (studies and reviews) as graphically presented in Fig. 2a, b

Topic	Important liquid biopsy-derived biomarkers	Number of publications	Supplementary Table	References
Neuroblastoma	*MYCN* and *ALK* amplification, CNAs (1p, 11q, 17q)	121	2	^51–123,138–142,144–149,152,153,235,236,357,380–411^
Renal tumors	CNAs (1q, 1p, 7q, 7p, 11p15, 16q, MYCN) and mutations (*TP53*, *MYCN*, *WT1*, and *CTNNB1*)	21	3	^9,106,139,140,141,148,235,236,178–183,190,412–417^
Germ cell tumors	miR-371-373, 302, and 367 clusters	7	4	^9,196,209,213–216^
Osteosarcoma	Heterogeneous profile (complex SVs in ctDNA, miRNA and lncRNA profiles, CTC assessment)	106	5	^9,106,139,140,148,149,235,236,232–234,237,238,240,241,243–245,247,263,306,335,336,418–500^
Ewing sarcoma	*FET-ETS* family fusion genes (most notably *EWSR1-FLI1*)	38	6	^9,106,122,123,139,140,145,146,148,149,236–238,263–269,271–276,306,321,335,336,501–508^
Rhabdomyosarcoma	*PAX3/7-FOXO1* fusion genes (ARMS only). Heterogeneous profile for ERMS (complex patient-specific SVs)	34	7	^9,106,122,139,140,141,145,146,148,149,236,237,268,289,296–309,335,336,501,509–511^
Non-rhabdomyosarcoma soft tissue sarcoma	Recurrent subtype specific fusion genes (*SS18-SSX* for SS and *EWSR1-WT1* for DSRCT)	9	8	^9,140,149,237,268,309,334–336^
Liver tumors	*CTNNB1* mutations and miRNA profiles	8	9	^9,106,235,343,346,347,349,350^
Reviews		61	10	^3,4,8,31,35,42,43,47,50,124–137,161,164,165,197,200,202,228,231,248–256,261,270,279,286,512–528^
Total unique publications		344		^3,4,8,9,31,35,42,43,47,50,51–123,124–137,138,139,140,141,142,144,145,146,147,148,149,152,153,161,164,165,178–183,190,196,197,200,202,209,213–216,228,231–234,235,236,237,238,240,241,243–245,247–256,261,263–276,279,286,289,296–309,321,334–336,343,346,347,349,350,357,380,381–528^

Per tumor type, important liquid biopsy-derived biomarkers, relevant studies, and their references are indicated. References for identified review articles are also provided. Studies that included pediatric patients for more than one tumor type are included in rows corresponding to each respective tumor type.

In this review, we provide a comprehensive overview of liquid biopsy-based biomarkers within the literature for the most frequent pediatric solid tumors, including neuroblastoma, renal tumors, germ cell tumors, osteosarcoma, Ewing sarcoma, rhabdomyosarcoma, non-rhabdomyosarcoma soft tissue sarcomas, and liver tumors. As such, hematological malignancies (leukemias and lymphomas) and central nervous system tumors were not included here.

## Comprehensive literature analysis

An overview of research articles on liquid biopsies in pediatric solid tumors was generated through an advanced search in PubMed (see Supplementary Table 1 for search parameters, selection criteria, and exclusion criteria). Generally, studies were included if they investigated liquid biopsy-based biomarkers in pediatric patients suffering from one or more solid tumor types within our selection. Review articles focusing on liquid biopsies in one or more of these pediatric tumor types were included as well. Articles (studies and reviews) focusing on liquid biopsies within adult tumors or pediatric tumors outside our selection were not included.

Following our literature search, we identified 344 unique publications (283 studies and 61 reviews). We provide a historical overview of these articles, as well as a historical overview of studies investigating liquid biopsy-based biomarkers in pediatric patients, per tumor type (Fig. 2a, b, respectively). Studies that included pediatric patients with more than one tumor type were included in each respective tumor type plot in Fig. 2b. Research on liquid biopsy-based biomarkers within pediatric solid tumors clearly started in the early 90 s, with many articles having been published over the last 10 years.

In the following chapters, per tumor type, we briefly describe the respective malignancy, followed by involved genetic aberrations and a review of the literature on liquid biopsy implementation. For each tumor type, we provide a comprehensive overview of studies that included pediatric patients of the respective tumor type (Supplementary Tables 2–9). Per individual study, we include the investigated liquid biopsy-based biomarkers, analytes, matrices, detection methods, number of pediatric patients included, and suggested application of the biomarker(s). The neuroblastoma and osteosarcoma chapters are the only exceptions due to the excessively high number of publications on these tumor types, and we provided more generic tables instead (focusing on ctDNA-derived biomarkers for neuroblastoma, and different liquid biopsy analytes for osteosarcoma). A summary of important liquid biopsy-derived biomarkers and references to the relevant studies, per tumor type, are provided in Table 1.

## Liquid biopsies in defined pediatric solid tumors

### Neuroblastoma

Neuroblastoma (NB) is the most common extracranial childhood tumor, accounting for roughly 8% of childhood malignancies^42–44^. NB originates from neural crest progenitor cells, and can establish anywhere along the sympathetic nervous system with the majority arising in the adrenal glands^45^. NB is highly heterogeneous, and clinical symptoms and presentation can vary from a benign mass to substantially metastasized disease^45^. NB is classified into low, intermediate, and high-risk (approx. 50%) groups based on several criteria^46^. These include age at diagnosis, tumor stage and differentiation, tumor histology and associated DNA ploidy, *MYCN* status, and chromosome 1p and 11q copy number status^42,47,48^. The median age of diagnosis is 18 months, with 40% of patients being diagnosed at infancy and 90% at <10 years of age^49^. The 5-year survival rate is highly dependent on the risk group, varying from >90% in the low-risk group to 40–50% for patients in the high-risk group^50^. Treatment depends on disease stage and includes simple observation, surgical resection, chemotherapy, radiation therapy, myeloablative chemotherapy with autologous stem cell reinfusion, and immunotherapy^45^.

NB is one of the few pediatric cancers where biomarkers are routinely used for diagnosis, prognostication, and therapeutic monitoring, including urine and serum-derived markers (neuron-specific enolase, lactate dehydrogenase, vanillylmandelic acid, and homovanillic acid)^47^. Nonetheless, these biomarkers lack sensitivity and specificity for accurate prognostication^42^. Also, NB diagnosis, risk-classification, therapeutic monitoring, and relapse detection still mostly relies on invasive tumor and bone marrow biopsies and imaging methods^43^. Over the last two decades, liquid biopsies (notably blood plasma and bone marrow) have increasingly been investigated as an alternative source for potential NB biomarkers (Fig. 2b and Table 1). ctDNA is the most investigated analyte (Supplementary Table 2). Furthermore, CTCs have been analyzed using detection of nucleosomes, mRNA, DNA, cfRNA (including miRNA), and EVs.^51–123^. Many of the studies investigating these analytes in NB have already been thoroughly reviewed elsewhere^3,4,8,31,34,35,42,43,47,50,124–137^. Therefore, we will focus on the most recent development for ctDNA analysis and implementation here.

ctDNA (being the tumor fraction of cfDNA) constitutes of 90–150 bp long fragments that are actively (in EVs) or passively (by necrosis or apoptosis) released from malignant cells [12]. cfDNA is rapidly cleared from circulation (half-life of minutes to 2.5 hours) making it an interesting biomarker for real-time tumor analysis [11]. NB displays particularly high ctDNA levels at diagnosis compared to other (pediatric and adult) cancer types including Wilms tumor, rhabdomyosarcoma, osteosarcoma, Ewing sarcoma, melanoma, renal cell carcinoma, hepatocellular carcinoma, medulloblastoma, glioma, and prostate, ovarian, colorectal, breast, thyroid, head and neck, pancreatic ductal, bladder, and gastroesophageal cancer^31,47,138,139,140^. Also, significant correlations among cfDNA concentration and tumor volume has been observed in NB patients, as well as higher cfDNA levels in metastatic NB versus localized NB^141,140^. Multiple studies demonstrated the suitability of plasma cfDNA content as a biomarker for NB risk-stratification, tumor load, monitoring of treatment response, and detection of MRD and relapse (Supplementary Table 2). Also, recent technological advances significantly facilitated ctDNA detection and analysis^4^. Novel methods such as droplet digital PCR (ddPCR) now allow rapid and cost-effective screening for multiple genetic targets despite small sample volumes and low ctDNA concentrations, as well as offering absolute quantification^42,142^. Important genetic NB targets that are detectable using ctDNA include (but are not limited to) gene amplifications (*MYCN* and *ALK*), chromosomal copy number changes (1p and 11q loss, and 17q gain), and epigenetic aberrations (*RASSF1A* and *DCR2* hypermethylation)^43,143^. A comprehensive overview of studies investigating ctDNA analysis in NB is provided in Supplementary Table 2. For example, Peitz et al.^142^ demonstrated two multiplex ddPCR protocols allowing reliable quantification of *MYCN* and *ALK* copy numbers in NB patients using cfDNA and matched tissue samples. Similarly, Shirai et al.^144^ combined *MYCN* copy number and loss of 11q heterozygosity for accurate diagnosis in NB patients using plasma-derived cfDNA with ddPCR. Then, Gelineau et al.^145^ recently described a case-series where cfDNA contributed to the successful diagnosis and prognostication in NB patients using ddPCR, qPCR, and reduced representation bisulphite sequencing of cell-free DNA (cfRRBS). Finally, van Zogchel et al.^146^ used targeted locus amplification and ddPCR to identify patient-specific targets in the cfDNA of NB patients. These markers tracked tumor burden, decreased during induction therapy, disappeared at complete remission, and re-appeared at relapse. Some markers were detectable in cfDNA before relapse detection by imaging or standard bone marrow evaluation, demonstrating suitability for monitoring NB burden, disease progression, MRD detection, and early relapse prediction. Next to ddPCR, chromosomal micro-arrays can also be used for the identification of patient-specific copy number profiles and intra-tumoral heterogeneity^42^. For example, López-Carrasco et al.^147^ showed improved detection of intra-tumor heterogeneity in NB patients when combining tumor biopsy and ctDNA analysis *versus* tumor biopsies alone, therefore providing more accurate genomic diagnosis, prognosis, and therapy options.

The emergence of advanced sequencing techniques now allows for targeted or genome-wide screening and sensitive detection of patient-specific genetic aberrations^4^. For example, Van Paemel et al.^148^ accurately diagnosed 67 NB cases out of 128 pediatric cancer patients by CNA profiling using shallow whole genome sequencing (sWGS) on paired plasma cfDNA and tissue DNA samples. Also, Cahn et al.^149^ performed molecular profiling of 324 genes in 12 NB patients for the identification of actionable targets using a commercially available platform (Foundation One Liquid CDx). They concluded that especially in refractory or relapsed NB patients, liquid biopsies are very suitable for obtaining molecular tumor information for guiding therapeutic decisions in a clinically relevant timeframe.

NB pathogenesis is also correlated with epigenetic aberrations, providing additional targets for NB detection in ctDNA^43^. As with many cancers, *RASSF1A* is often hypermethylated in NB^150^. van Zogchel et al.^141^ found hypermethylated *RASSF1A* in plasma of 41 of 42 patients with metastatic NB. Levels decreased during therapy and recurred at relapse, demonstrating its potential value as a prognostic biomarker after diagnosis. Next, enzymatic methyl-sequencing (EM-seq) emerged as a new method for methylome analysis outperforming the classic bisulphite sequencing method^151^. Two studies recently demonstrated the feasibility of EM-seq for whole methylome analysis from ctDNA in NB patients^152,153^. For example, Trinidad et al.^152^ revealed prognostic actionable targets in high-risk 11q deleted NB and a DNA-methylation biomarker profile for disease monitoring using very low input cfDNA.

Taken together, conducted studies including the examples described above demonstrate the clinical potential of liquid biopsies (notably ctDNA) for implementation in NB, including diagnosis, tumor burden evaluation, treatment efficacy, intra-tumor heterogeneity, and the detection of MRD and relapse (Supplementary Table 2). However, several obstacles need to be tackled before clinical implementation can be realized including biomarker validation in prospective multi-center trials with harmonized and standardized protocols from sample collection to final data analysis^8,31,125,154^. An elaboration of these considerations is given in the ‘challenges and requirements for clinical implementation’ chapter. Importantly, liquid biopsy sampling and analyses are included in the ongoing SIOPEN-HR-NBL2 clinical trial (NCT04221035)^155^. Furthermore, Liquid biopsies will be used to facilitate clinical decision making in the EU-funded international MONALISA trial (EU- Grant Agreement number 101137028).

### Renal tumors

Renal tumors comprise 5% of all pediatric cancers^156^. Important subtypes include Wilms tumor (WT)/ nephroblastoma, clear cell sarcoma of the kidney (CCSK), malignant rhabdoid tumor of the kidney (MRTK), pediatric renal cell carcinoma (RCC), and congenital mesoblastic nephroma (CMN)^157–160^. These subtypes display high heterogeneity in histology, genetic aberrations, and malignant potential^157^. WT is the most common type comprising 80-90% of cases, and hence will be the focus here^161–163^.

WT is an embryonal cancer containing undifferentiated blastemal, stromal, and epithelial elements, similar as during fetal development^164^. It is one of the most common malignant childhood tumors with roughly 95% occurring in patients below the age of 10^165,166^. The Children’s Oncology Group (COG) and The International Society of Pediatric Oncology-Renal Tumor Study Group (SIOP-RTSG) protocols comprise the two major directions of WT management, which mostly differ in surgery timing and consequent stratification for chemotherapy and radiotherapy,^167,168^. Outcomes for WT patients have significantly improved in recent decades with a five-year survival rate from 75% in 1975-1979 to 90% in 2003–2009^169,170^. Nonetheless, approximately 15% of patients eventually relapse with an OS rate of 50%^171^. These relapses occur mainly in the (stage and histology-based) intermediate-risk subgroups, and are often not recognized^172^. Also, although fine needle biopsies are considered rather safe with respect to tumor rupture risk, strict criteria for such diagnostic procedures are currently being used^4,31,173^. Consequently, a lack of histological confirmation could potentially lead to misdiagnosis and hence suboptimal treatment, especially in non-HR WT patients^135^. Finally, WT displays a high degree of intra-tumor genetic heterogeneity^174,175^. Hence, liquid biopsies could aid in renal tumor diagnosis, subtype identification, treatment guidance, risk-stratification, assessment of intra-tumor heterogeneity, and early relapse detection^161,165,174^. A comprehensive overview of studies involving analysis of liquid biopsy-based biomarkers in pediatric renal tumors is depicted in Supplementary Table 3.

Among pediatric solid tumors, WT presents with relatively high ctDNA concentrations, further demonstrating the potential for liquid biopsy application^139,141,140^. For example, Ruas et al.^140^ and Klega et al.^139^ found the second highest (following NB) plasma cfDNA levels in WT patients versus Ewing sarcoma, rhabdomyosarcoma, leiomyosarcoma, osteosarcoma, and benign teratoma patients. Next, van Zogchel et al.^141^ measured significantly higher ctDNA levels in WT (and NB) patients *versus* pediatric controls, while this difference was not significant in patients with rhabdomyosarcoma, lymphoma, or CNS tumors. Also, despite its genetic heterogeneity, WT displays several recurrent aberrations^161,165^. COG identified 1p loss, 1q gain, and 16q loss, as adverse prognostic factors, which have now been included for risk-stratification in the USA^176,177^. Also, 1q gain and 11p15 loss have been associated with relapse and death in WT, making these CNAs interesting targets for early relapse detection^165^. Indeed, several studies investigated plasma or serum-derived ctDNA for WT biomarkers including CNAs (1q, 7q, and *MYCN* gain, and loss of 1p, 7p, 11p15, and 16q), but also somatic mutations (including *TP53*, *MYCN*, *WT1*, and *CTNNB1*) and genomic methylation (Supplementary Table 3). A recent report from the COG prospective AREN0533 trial by Madanat-Harjuoja et al.^178^ used NGS approaches for ctDNA detection patients with stage III or IV WT. They detected ctDNA in serum of 41/50 patients and observed a trend toward worse event-free survival (EFS) for patients with detectable ctDNA (80% *versus* 100%). In contrast, none of the patients with undetectable ctDNA in serum relapsed and these all survived. Finally, they identified several CNAs (1q, 1p, and 16q) and single nucleotide variants (SNVs) in ctDNA of which some were not detectable in tumor biopsies^178^. Concurrently, van Paemel et al.^148^ identified several chromosomal aberrations (including the prognostic marker 1q gain) in cfDNA of WT patients that were not detectable in matched tissue DNA. Therefore, ctDNA analysis could potentially identify subclonal copy-numbers that might be missed when profiling a single tumor tissue at a specific site^161^. Still, other studies indicated that some genetic aberrations (CNAs and SNVs) in tumor biopsies were not detectable in corresponding cfDNA in some WT patients, suggesting the need for larger cohort studies that include harmonized methods for ctDNA isolation and detection for biomarker validation^179,180^.

Next to ctDNA alternative peripheral blood-derived analytes have been identified as potential WT biomarkers (Supplementary Table 3). For example, He et al.^181^ measured elevated levels of XIST, a lncRNA, in 49 WT patients using microarrays and RT-qPCR, and significantly correlated XIST upregulation with tumor staging and shorter survival time. Next, Ludwig et al.^182^ demonstrated a serum miRNA panel (most notably miR-100-50 and miR-130b-3p) that differentiated 43 WT patients from 13 healthy controls with an accuracy, sensitivity, and specificity of 79.6%, 69.2%, and 90.0%, respectively. Similarly, Schmitt et al.^183^ measured an elevated panel miRNA signature in 43 WT patients *versus* 19 healthy controls, but did not observe a significant difference in signature between patients prior and after chemotherapy. An elaborate description and explanation of miRNAs is provided in the germ cell tumor chapter.

Urine is increasingly being investigated as an alternative liquid biopsy matrix for renal tumor detection^184–186^. Especially renal tumor proximity, facilitation for serial-sampling, and the non-invasive nature make urine an interesting alternative^27,180^. Indeed, there have been several efforts investigating urine-based biomarkers in pediatric renal tumors (Supplementary Table 3). Early studies in the 90 s showed elevated levels of hyaluronidase, hyaluronic acid, and basic fibroblast growth factor in urine of WT patients^187–189^. More recently, Ortiz et al.^190^ identified multiple urinary protein biomarkers in 139 pediatric patients with different types of pediatric renal tumors. Particularly, high urinary prohibitin concentrations at diagnosis were significantly associated with relapse in WT patients. Also, the earlier described COG study by Madanat-Harjuoja et al.^178^ detected ctDNA in urine of WT patients, however, in a significantly smaller sample group *versus* matched serum samples (13/50 *versus* 41/50). Hence, more sensitive assays or larger sample volumes might be required for urine-based liquid biopsies.

Taken together, ctDNA detection, subsequent genetic targets, and other analytes are promising markers for WT risk-stratification and prognostication but require validation in larger cohorts^178^. Also, there is limited data available on serially collected liquid biopsy samples for monitoring treatment response and follow-up^161^. Finally, no biomarkers for reliable differentiation among different pediatric renal tumor subtypes have been identified so far. This is important to prevent misdiagnosis and suboptimal treatment for patients that are being treated for WT without histological confirmation^3,31,135^. Peripheral blood and urine liquid biopsy sampling, and subsequent ctDNA, miRNA, and proteomic analyses are therefore pursued in the ongoing SIOP-RTSG-2016 UMBRELLA clinical trial^158^.

### Germ cell tumors

Germ cell tumors (GCTs) arise from primordial germ cells and derivatives during embryogenesis. Most GCTs develop in the gonads or along the midline structures of the body, but other locations including the central nervous system are common as well^191,192^. Pediatric GCTs are classified in two types depending on age group and histology. Prepubertal type I GCTs are mostly present in children aged 0–4 and may present as yolk sac tumor (YST), (generally) benign teratoma, or mixtures of these elements^193^. Within this group, GCTs are generally rare representing only 3% of all cancers^5^. Post-pubertal type II GCTs commonly arise from puberty to early adulthood and are represented by a wider range of histologies and subtypes (germinomatous GCTs and non-germinomatous GCTs), almost always being malignant^192,193^. There, pediatric GCTs are more common (accounting for 13% of all tumors for adolescents aged 15–19)^5^.

The serum tumor markers beta-human chorionic gonadotropin (β-hCG), alpha-fetoprotein (AFP), and lactate dehydrogenase (LDH) are clinical standards for GCT diagnosis and follow-up^194^. β-hCG and AFP are commonly linked to choriocarcinoma and (type I and II) YST, respectively^194,195^. In contrast, LDH is the least specific and rather used as indicator of cell turnover and burden of disease^194^. Generally, the lack of specificity and low sensitivity of these serum markers limits utility for histological subtype discrimination and relapse detection^196–198^. Therefore, there is a need for alternative biomarkers allowing more sensitive and specific GCT detection regardless of age, (malignant) subtype, and anatomical location without elevation in benign-GCTs and non-GCT tumors^199^. miRNAs are a promising alternative to conventional serum markers^200^. These small non-coding RNA molecules of 20-24 bases are involved in many biological pathways within multicellular organisms^201^. Being regulators of cell proliferation and differentiation, they influence numerous cancer-relevant processes such as proliferation, cell cycle control, apoptosis, cell differentiation, migration, and metabolism^201^. There, dependent of their mRNA target(s), they can function either as oncogenes or tumor suppressors^197^. Also, miRNA molecules are sensitive, specific, stable, and require low-cost equipment for isolation and detection in blood (serum and plasma), making them interesting liquid biopsy biomarkers for cancer detection and pathogenesis^200^.

The significance of miRNAs as alternative GCT biomarkers has been emerging in the last two decades^200,202–205^. Voorhoeve et al.^204^ were the first to demonstrate this by identifying miR-372 and miR-373 as potential oncogenes in testicular GCTs (TGCTs) through intervention with the p53 pathway in cell lines. Following a high throughput screening of 156 miRNAs in human GCT material, involvement of miRNA 371-373 clusters in type II malignant GCTs (MGCTs) was confirmed^206,207^. Later, Palmer et al.^208^ demonstrated overexpression of miR-371-373 and miR-302-367 clusters in all MGCTs, regardless of age, site, and histological subtype. Following demonstrated isolation and detection from blood^199,209–211^, this miRNA panel has hence been shown to be a highly sensitive and specific liquid biopsy based approach for MGCT detection^200,202,212^.

Murray et al.^209^ were the first to document involvement of liquid biopsy-derived miRNAs in pediatric GCTs. Using a multiplex RT-qPCR approach, they measured elevated miRNA levels of the 371-373 and 302 clusters in a 4-year-old boy with a YST at diagnosis and baseline levels during clinical follow-up, with parallel AFP kinetics. Subsequently and using exclusive pediatric cohorts, they demonstrated suitability of specific miRNAs (particularly miR-371a-3p) for pediatric MGCT diagnosis at different anatomical locations (intra and extracranial) and subtypes (choriocarcinoma, YST, and mixed MGCTs (MMGCTs))^196,213,214^. These miRNAs could also detect MGCT relapse while AFP and β-hCG levels were still below threshold levels for diagnosis^213,214^. Finally, they allowed discrimination of intracranial MGCT (iMGCT) from intracranial non-GCT tumors^196^. Concurrently, Schonberger et al.^215^ recently demonstrated suitability of these miRNA clusters for iMGCT diagnosis as well. Finally, Saliyeva et al.^216^ recently measured elevated levels of miR-302/367 and 371-373 clusters in 20 pediatric patients with various MGCTs. There, miR-371,372,373,367, and 302d levels were significantly higher in GCT patients (various subtypes) *versus* controls. miRNA levels of the 371-373 cluster were highest in patients with pure dysgerminoma *versus* non-germinomatous GCTs. MiR-367 levels were elevated regardless of morphological GCT type, but highest in patients with stage III-IV disease^216^. A comprehensive overview of studies involving liquid biopsy analysis in pediatric GCT patients is depicted in Supplementary Table 4.

The studies discussed above support the use of the miR-371-373, 302, and 367 clusters as sensitive and specific liquid biopsy biomarkers for pediatric GCT diagnosis and monitoring. However, validation in large pediatric cohorts is required as the provided studies only included small patient groups since most liquid biopsy-derived miRNAs studies have focused on post-pubertal type II TGCTs^197,213,214^. This is especially relevant for prepubertal type I GCTs, which represent a distinct disease *versus* type II GCTs that seem histologically and biologically similar among adolescents and adults^193^. Two currently running clinical trials that include liquid biopsies and focus on pediatric GCTs include the MAKEI-V and MAGIC trials^217,218^.

While miRNAs from the specified clusters have shown to be involved in all MGCTs, they fail to identify the teratoma subtype (both type I and II)^202,208,219^. Teratomas consist of a disordered mixture of differentiated cell types possibly from all three somatic germ cell layers. They are generally benign (type I), since their formation is the result of improper developmental differentiation rather than via an oncogenetic driver^193^. Nonetheless, they are therapeutically relevant as they are generally resistant to chemotherapy and surgical excision is the only therapeutic option. Therefore, they need to be distinguished from MGCTs where classical serum markers are generally not useful for confirming this GCT subtype^219^. Lobo et al.^219^ combined miR-371a-3p and hypermethylated *RASSF1A* detection, of which the latter is a common feature in several solid malignancies. This combined liquid biopsy panel allowed for 100% sensitivity in detecting diverse (young) adult TGCT subtypes, including (type II) teratoma. Then, Nappi et al.^220^ suggested the combination of miR-371a-3p and miR-375-5p to be highly accurate for (type II) teratoma detection. However, the level of informativity of miR-375 has been questioned in other papers^216,221–225^. Future studies should include large pediatric cohorts with type I prepubertal GCTs to investigate whether this combined panel also allows for prepubertal teratoma detection.

### Sarcomas

#### Osteosarcoma

Osteosarcoma is the most common bone tumor, primarily affecting children, teens, and young adults, and makes up 2% of cancers in children aged 0–14, and 3% in teens aged 15–19^5,226,227^. It arises from a malignant transformation of mesenchymal cells producing osteoid and/ or immature bone, and can present in any bone of the body with the most common sites being the metaphyses of long bones (distal femur, proximal tibia, and the proximal humerus)^228,229^. Standard treatment of patients with high-grade osteosarcoma (majority of patients) involves multiagent induction chemotherapy followed by surgical resection for local tumor control with focal therapy directed to metastatic sites (10–20% of patients have metastasis at the time of initial diagnosis)^34,226^. Osteosarcoma incidence has been relatively stable over the last 40–50 years with a reduction in mortality rates, primarily due to the introduction of multiagent chemotherapy^226^. Nonetheless, metastasis and chemoresistance are still a serious problem^230^. Metastases are detected in roughly 10–40% of osteosarcoma patients (higher percentage in low and middle-income countries), and patients with metastasized disease have a 5-year survival rate below 30%^230^.

Next to the advantages of liquid biopsies addressed before, implementation within bone tumors has additional value since bone tumor biopsies can be dangerous (e.g., when in proximity to the spinal cord) and difficult to collect^231^. Here, we will focus on the most described liquid biopsy-based analytes for osteosarcoma, being ctDNA, CTCs, miRNAs, and lncRNAs. A comprehensive overview of liquid biopsy-based analytes (including less-studied examples) and involved studies is provided in Supplementary Table 5.

Osteosarcoma displays high genomic complexity, instability and heterogeneity, resulting in a high number of SVs that can cause fusion genes, alternative splicing, CNAs, and gene expression dysregulations^31,130,228,231–233^. This genomic heterogeneity and lack of recurrent genetic aberrations might limit the implementation of ctDNA targeted assays such as ddPCR within osteosarcoma *versus* other pediatric tumors^3,234^. Nonetheless, several studies detected ctDNA in peripheral blood of osteosarcoma patients at diagnosis and during follow-up, and demonstrated a correlation between ctDNA levels, treatment response, and survival (Supplementary Table 5). Most studies utilized NGS approaches for identification of the complex SVs associated with osteosarcoma^139,148,232,235,236,237,238^. For example, Shulman et al.^238^ performed ultra low pass-WGS (ULP-WGS) for ctDNA detection in 72 patients with primary localized osteosarcoma. Although statistically insignificant, ctDNA was detectable at diagnosis in 41/72 of these patients, and increased ctDNA levels were correlated with increased risk of death. DNA methylation is an alternative marker for ctDNA detection that could be particularly interesting for osteosarcoma as methylation changes are often retained through tumor evolution, and are only moderately confounded by tumor genetic heterogeneity^239^. Lyskjær et al.^234^ employed methylation-specific ddPCR for the discovery and validation of methylation-based biomarkers for ctDNA detection in 72 osteosarcoma patients. They detected ctDNA in 50/72 or 29/72 of pre-operative plasma samples (based on one or two marker thresholds). Also, ctDNA was detected in 5/17 post-operative plasma samples, which in four cases was associated with or preceded relapse. Both pre-operative cfDNA levels and ctDNA detection independently correlated significantly with OS^234^. Finally, van Paemel et al.^236^ used cfRRBS for methylome profiling for the correct diagnosis of 4/4 osteosarcoma cases within a pool of 60 samples derived from patients harboring different solid tumors.

As tumor recurrence and metastasis are the leading causes of death in osteosarcoma patients, there is a particular need for biomarkers assessing tumor clonal evolution, prediction of treatment resistance, and metastatic potential and/ or early relapse detection^240,241^. CTCs can be an additional sensitive target for this purpose. CTCs circulate freely in the bloodstream with genetic resemblance to their tumor origin^3^. They constitute a very small fraction of cells in the bloodstream and require validated methods for detection, enrichment, and enumeration^3^. CTC sampling provides live information regarding disseminated tumor cell heterogeneity, and has already been implemented in clinical practice for metastatic tumor prognosis of several adult tumors such as colorectal, breast, and prostate cancer^231,242^. CTCs have been described as potential predictive and prognostic biomarkers for osteosarcoma metastasis^240,241,243–245^. Dai et al.^243^ recently investigated CTCs using the Canpatrol CTC detection platform in 50 osteosarcoma patients (23 children and 27 adults, 34 with localized disease and 16 with metastatic disease). They detected CTCs in 43/50 patients and counts were significantly higher in Enneking Stage III (regional or distant metastasis) patients *versus* IIB (no metastasis) patients^243,246^. Also, CTC detection with positive Insulin-like growth factor mRNA-binding protein 3 signal best diagnosed osteosarcoma metastasis (87.5% sensitivity, 82.4% specificity). Finally, serial monitoring in one patient suggested that total CTCs were associated with disease progression^243^. Future studies focusing on CTC detection and implementation in osteosarcoma should include large cohorts involving serial sampling during treatment and follow-up. This will allow for dynamic CTC monitoring to assess chemotherapy response and timely detection of recurrence or metastasis, as well as investigate relationships among CTC detection and survival^240,243^.

The potential role of miRNAs and lncRNAs as biomarkers for osteosarcoma has been extensively studied over the last 10 years, and several studies showed promising diagnostic and prognostic performance (Supplementary Table 5). For example, Xie et al.^247^ found significantly higher circulating miR-26a-5p levels in serum of 243 osteosarcoma patients *versus* 96 healthy controls. Patients with high miR-26a-5p levels also had poorer OS *versus* those with low levels, and multivariate analysis showed miR-26a-5p to be an independent osteosarcoma risk factor^247^. Most other (older) miRNA and lncRNA studies for osteosarcoma have already been extensively reviewed elsewhere^34,129,137,228,231,248–256^. Importantly, despite the extensive number of publications and promising performance of these RNAs in osteosarcoma, there has been little overlap in candidates and consistency in results among studies. Also, several investigated biomarkers are not specific for osteosarcoma (such as miR-21 and miR-20a)^250^. Finally, most studies were performed in Asia (mostly China), thereby limiting the translation of biomarker performance for osteosarcoma to other countries and regions^255^. This, again, demonstrates the need for large-scale, prospective, international multi-center clinical trials. Due to osteosarcoma heterogeneity, several methods and targets should be included in such trials including ctDNA quantification and subsequent broad sequencing approaches, CTC enumeration and profiling, and RNA (miRNA and lncRNA) profiling. Combining these different analyses hopefully enables development of a sensitive and specific biomarker panel for osteosarcoma.

#### Ewing sarcoma

Ewing sarcoma (EWS) is a malignant tumor predominantly occurring in bone (mostly pelvis, femur, tibia, and ribs), and in soft tissues (thoracic wall, gluteal muscle, pleural cavities, and cervical muscles)^257^. Although EWS origin is a matter of debate, it is thought to involve neural crest cells and/ or mesenchymal stem cells.^258–260^. Despite being rare, EWS is the second most common bone tumor in children, adolescents, and young adults (following osteosarcoma) comprising roughly 2% of childhood malignancies^44,257,261^. Peak incidence occurs around 15 years of age, and occurrence in older adults is very rare^257,260^. EWS is highly aggressive and survival rate varies from 70-80% for patients with localized disease to approximately 30% for those with metastasis^257^. Standard of care consists of a multimodal treatment regimen including multi-agent chemotherapy, surgical resection, and radiotherapy^231^.

EWS is characterized by chromosomal translocations yielding fusion genes and corresponding fusion proteins that promote tumorigenesis^257,262^. These fusion proteins contain a member of the FET family of proteins (most commonly) being RNA-binding proteins involved in transcription and splicing, together with different members of the E26 transformation-specific (ETS) family of transcription factors, which are involved in cell proliferation, differentiation, cell-cycle control, angiogenesis, and apoptosis^257,261^. 90% of EWS cases are driven by a t(11;22)(q24;q12) chromosomal translocation, leading to the *EWSR1-FLI1* fusion gene^154,257,261–263^. *EWSR1-ERG* is the next most common fusion complex (5-10%) and other *EWSR1* gene fusions (with *ETV1*, *ETV5*, or *FEV1*) are rare (<1% of total cases)^261^. Similarly, some variant fusions between other *FET* gene families (*FUS* and *TAF15*) with *ETS* family members have been described^257^. Apart from these fusion complexes, EWS is a relatively genetically silent cancer with few recurrent mutations or CNAs^31^.

Fusion genes such as *EWSR1-FLI1* provide a clinically relevant liquid biopsy-based target for EWS detection and monitoring^231^. Initial efforts investigating this utilized RT-PCR approaches for fusion gene detection in CTC-derived RNA in EWS patients^264–269^. While fusion transcripts were indeed detectable, the clinical significance and prognostic value of these findings were inconclusive among studies^270^. During the last 20 years, focus shifted from CTC detection to ctDNA analysis. Technological advances such as ddPCR allowing accurate nucleic acid detection and analysis recently rekindled interest in liquid biopsy implementation in EWS^270^. Indeed, over the last 7 years, many novel studies started investigating peripheral blood or bone marrow derived *EWSR1* fusion genes as biomarker for pediatric EWS (Supplementary Table 6). For example, Krumbholz et al.^271^ evaluated the predictive and prognostic value of ctDNA in 102 EWS patients using *EWSR1* fusion primers and probes for sensitive and specific ctDNA detection in plasma by ddPCR. Pretreatment ctDNA copy numbers were correlated with EFS and OS, and ctDNA levels decreased below limit of detection (LOD) after two blocks of induction chemotherapy. Also, persistence of ctDNA after chemotherapy was a strong predictor of poor outcomes and ctDNA levels correlated well with clinical risk factors (tumor size, osseous or pelvic location, metastasis, and age at diagnosis) suggesting suitability for early prediction of treatment response and outcome^271^. Next, Schmidkonz et al.^272^ used ddPCR for ctDNA detection based on individual *EWSR1-FLI1* and *EWSR1-ERG* fusion sequences. They found a significant correlation between ctDNA signals and tomography signaling (^18^F-FDG-PET/CT), for diagnosis, treatment response, and relapse detection in 20 EWS patients.

Next to ddPCR approaches, sequencing-based methods also enabled ctDNA detection and analysis including identification of precise fusion breakpoints and novel DNA rearrangements (Supplementary Table 6). For example, Shah et al.^237^ used a deep sequencing approach for common translocation detection in plasma from patients with common sarcomas including EWS. They found that ctDNA levels correlated with metastatic status and clinical response, and quantified significant ctDNA levels several months before relapse was clinically detected by imaging. More recently, Christodoulou et al.^9^ demonstrated a hybridization-based capture panel targeting *EWSR1* fusions that correctly identified 10/12 EWS patients within a pool of 73 pediatric solid tumor patients. Finally, Seidel et al.^273^ used WGS for the identification of exact *EWS-FLI1* fusion gene breakpoints followed by ddPCR for specific ctDNA detection. They showed that ctDNA detection in peripheral blood of 6 pediatric EWS patients allows for real-time monitoring of MRD activity, although patients displayed high variability in ctDNA signal among individuals.

Next to ctDNA, *EWSR1* fusion genes can also be detected in ctRNA and small extracellular vesicles (sEVs) using ddPCR and RT-qPCR^274–276^. However, a comparison by Bodlak et al.^276^ suggested that ctRNA signals seem to be lower and statistically less reliable *versus* ctDNA detection. A comprehensive overview of relevant studies focusing on fusion gene detection in liquid biopsies for pediatric EWS is depicted in Supplementary Table 6.

Taken together, common *EWSR1* fusion gene detection provides a clinically relevant genetic biomarker panel for EWS detection. The studies mentioned here demonstrate the potential for several clinical applications including EWS diagnosis, early risk-stratification and prognosis, monitoring of therapy and clonal evolution, follow-up, MRD detection, and relapse prediction (Supplementary Table 6). The frequency of *EWSR1* fusion gene occurrence in EWS supports potential clinical use for most patients^130^. Also, experimental studies suggest that EWS tumor cells are dependent on such fusion genes reducing the risk for target loss by clonal evolution during treatment^31,262,277,278^. Hence, ctDNA quantification and analysis using ddPCR and deep NGS approaches targeting the characteristic fusion genes of EWS should be the main focus of future large cohort studies for validation of these potential EWS-specific liquid biopsy-based biomarkers^279^. Given the poor prognosis of EWS patients with metastatic or recurrent disease, risk-stratification is a particularly important target for improving outcome. Using ddPCR for fusion gene detection requires design of patient-specific primer-probe sets, which takes time (approx. 2 weeks)^271^. Hence, such assays must be developed in a clinically relevant timeframe for informing risk-stratification. After development, assay implementation for follow-up can be done in a faster timeframe^271^. The international INTER-EWING1 and German iEuroEwing studies are examples of EWS clinical trials that include liquid biopsy analyses^280,281^.

#### Rhabdomyosarcoma

Rhabdomyosarcoma (RMS) is the most common soft tissue tumor in children accounting for roughly 4.5% of all pediatric cancer cases^282,283^. It is the third most common extracranial solid childhood tumor following NB and WT, and most cases are diagnosed in children below the age of 6^283,284^. The most common sites of primary disease include the head and neck region, the genitourinary tract, and the extremities^283^. RMS treatment generally involves systemic treatment with intensive chemotherapy, and local surgery and/or radiotherapy^285^. Development and refinement of multimodal treatment regimens significantly improved pediatric RMS survival^282^. Nonetheless, survival rate depends heavily on the extent of disease at initial diagnosis, and survival for patients with metastatic or relapsed disease remains poor^286,287^. There are two RMS subtypes, being fusion gene-positive RMS (FP-RMS) and fusion gene-negative RMS (FN-RMS), depending on presence of *PAX3-FOXO1* or *PAX7-FOXO1* (hereafter referred to as *PAX3/7-FOXO1*) fusion genes caused by t(2;13)(q35;14) and t(1;13)(p36;q14) chromosomal translocations, respectively^2,34,129,286,288^. These characteristic fusion genes and subsequently fusion proteins drive the malignant phenotypes of FP-RMS tumors^286^. FP-RMS has alveolar histology, appearing histologically similar to pulmonary parenchyma^129^. In contrast to FP-RMS, the genetic landscape of FN-RMS is heterogeneous and does not involve specific chimeric genes^285^. FN-RMS tumors often include epigenetic modifications, various CNAs (e.g., chromosomal 2, 8, 12, 13 gain and 11p15.5 loss), or gene translocations/ mutations (e.g., *TFCP2, VGLL2, MYOD1, NCOA2, DICER1*)^286,289–291^. FN-RMS generally has embryonal histology, being composed of cells resembling immature skeletal myoblasts, although FN-RMS cases that display alveolar histology also exist^129,292^. Patients with FP-RMS have a worse prognosis than those with FN-RMS^2^. Consequently, *FOXO1* status has been identified as prognostic risk factor for RMS by the COG and The European pediatric Soft Tissue Sarcoma Group (EpSSG)^293–295^. Historically, RMS main types were discriminated based on histology, being alveolar RMS (ARMS) (20-25% of RMS cases) and embryonal RMS (ERMS) (70-75% of cases), instead of FP-RMS and FN-RMS^2,282^. Many of the studies reviewed here still adhered to this classical differentiation. As such, we use this terminology from here on as well.

Early studies in the 2000s that investigated liquid biopsy-derived biomarkers for RMS mostly focused on RNA-based *MYOD1*, *MYOG*, and *α/γACHR* transcripts^122,296–299^ (Fig. 2b and Supplementary Table 7). During the last five years, liquid biopsies in RMS have regained attention. The *PAX3/7-FOXO1* translocations are very well-suited as targets for detection of liquid biopsy-based biomarkers. Indeed, these fusion genes have demonstrated their potential in several liquid biopsy targets including ctDNA, CTC-derived mRNA, and cfRNA (Supplementary Table 7). To illustrate the potential of these fusion genes for RMS detection, Klega et al.^139^ used targeted sequencing and WGS for the detection of *PAX3-FOXO1* rearrangements in plasma-derived cfDNA from 7 ARMS patients. ctDNA changes corresponded with therapy response, with ctDNA often becoming undetectable at the start of the second chemotherapy cycle. Given that ERMS is the most frequent RMS type, there is an urgent need for biomarkers to identify and monitor this subtype in liquid biopsies. However, considering the heterogeneous genetic landscape of ERMS, this has been quite challenging so far ^286^. A comprehensive overview of studies focusing on liquid biopsy identification in pediatric RMS is indicated in Supplementary Table 7. Here, we will focus on recent studies that included the largest patient populations.

RMS-derived ctDNA can be detected by targeting tumor specific CNAs, patient-specific translocation breakpoints, CNVs, and/ or SNVs with different methods (Supplementary Table 7). For example, Ruhen et al.^300^ utilized ddPCR, panel sequencing, and whole exome sequencing (WES) for ctDNA detection in 28 RMS patients. ctDNA was detected in 21/25 pre-treatment plasma samples, and ctDNA levels at diagnosis were significantly higher in patients with unfavorable tumor sites, positive nodal status, and metastasis. Also, in serial plasma samples, ctDNA level fluctuations corresponded to treatment response^300^. More recently, Abbou et al.^301^ used ULP-WGS and targeted sequencing for ctDNA detection in 124 RMS patients. ctDNA was detectable in 40 cases, and patients with detectable ctDNA at diagnosis had significantly worse outcomes *versus* patients without detectable ctDNA (OS, 33.3% *versus* 83.2% and 39.2% *versus* 75%, depending on the subgroup)^301^. Next, Lak et al.^302^ employed ddPCR, shWGS, and cfRRBS for ctDNA analysis including methylated *RASSF1A* detection in 57 RMS patients. ctDNA was detected in 39/57 RMS patients at diagnosis, and most samples scored negative after the first course of chemotherapy. This transition is consistent with an earlier described study by Klega et al.^139^ Methylated *RASSF1A* was specifically detected in 21 of 57 patients and its presence significantly correlated with poor outcome (5-year EFS of 46.2% *versus* 84.9%). This prognostic effect of methylated *RASSF1A* was most apparent in patients with metastatic disease. Also, cfRRBS allowed for correct classification of the ERMS subtype in 21 out of 25 samples^302^. The potential of cfRRBS for pediatric solid tumor type classification has already been described earlier by van Paemel et al.^236^. There, they correctly classified RMS in 14 out of 17 cases (within a pool of 60 samples from a pediatric population with different solid tumors). ERMS and ARMS subclass identification was correct in 13 out of 14 cases.

Next to ctDNA, other analytes including CTCs, mRNA, miRNAs, exosomes, circulating protein, and monocytes have been suggested as liquid biopsy-based biomarkers for RMS (Supplementary Table 7). For example, Poli et al.^303^ measured significantly higher IGFBP2 protein and corresponding autoantibodies in 114 RMS patients *versus* healthy controls. IGFBP2 protein also identified metastatic patients with worse EFS, and both IGFBP2 and antibodies negatively correlated with OS^303^. Then, Lak et al.^289^ developed an RNA panel for detection of RMS-specific mRNA from the cellular fraction of blood and bone marrow, using multiplexed RT-qPCR in 99 RMS patients. Metastatic disease, detected by mRNA significantly outperformed standard of care testing, and the five-year OS was 54.8% for 33/99 RNA-positive patients *versus* 93.7% for 66/99 RNA-negative patients. When MRNA and methylated *RASSF1A* were combined in paired samples, the five-year OS was 100% *versus* 36% for double negative and double positive samples, respectively^302^. Finally, Urla et al.^304^ investigated expression of TKTL1 and Apo10 epitopes on monocytes in 29 RMS patients. Both epitopes scored positive in 96.5% of RMS patients and negative in the control population.

Taken together, the studies indicated in Supplementary Table 7 including those mentioned above demonstrate the potential of liquid biopsies for RMS diagnosis and risk-stratification, subtype identification, therapy monitoring, and outcome prediction. Given that survival in patients with metastatic or relapsed RMS is low, there is a particular need for more sensitive prognostic monitoring tools for response monitoring and relapse surveillance^286,300,301,305^. While several RMS studies involved serial liquid biopsy sampling including relapse samples, with some explicitly elaborating on relapse detection prior to confirmation using standard methods, population cohorts remain scarce^146,289,300,302,306–309^. Future trials should focus on detection of *PAX3/7-FOXO1* fusion genes using ddPCR and deep targeted NGS approaches. For PAX3/7-FOXO1 negative patients, broader sequencing approaches should be applied for identification of patient-specific SVs. Two currently running large-scale international trials (FaR-RMS and ARST1431) are including liquid biopsy studies and might offer further insight in the clinical and predictive value of these biomarkers^310,311^.

#### NRSTS

Non-rhabdomyosarcoma soft tissue sarcomas (NRSTS) constitute a heterogenous group of malignant extraskeletal mesenchymal tumors^312^. NRSTS includes over 35 distinct histologies, many of which are exceedingly rare^313^. Incidence of pediatric NRSTS types vary by age, as some typically occur in small children (e.g., infantile fibrosarcoma and malignant rhabdoid tumors), others in adolescents and young adults (e.g., synovial sarcoma (SS)), and some being more common in adults and rare in children (e.g., liposarcoma and leiomyosarcoma)^314,315^. Generally, NRSTS survival for patients with metastatic disease remains low^316,317^. For example, a report from the COG investigating pediatric SS (the most common NRSTS type) stated an OS of 97.7% and 84.9–89.5% for low and intermediate-risk patients, respectively^317^. In contrast, patients with metastatic disease (high-risk) only had an OS of 12.5%. In addition, a recent study from the European pediatric Soft tissue sarcoma Study Group reported a 3-year EFS and OS of 15.4% and 34.9%, respectively, for pediatric patients suffering from metastatic NRSTS^318^. This highlights the clinical value for liquid biopsy approaches for MRD detection as early prediction for metastatic disease^319^.

Gene fusions have been described as driver events in many NRSTS, of which most are recurrent in the same histologic subtype and, therefore, may serve as potential targets for liquid biopsy-based detection^320^. Indeed, some studies utilized this approach for SS (*SS18-SSX* fusions) and desmoplastic small round cell tumor (DSRCT) (*EWSR1-WT1* fusions) detection^237,268,309,321–323^. Nonetheless, studies investigating liquid biopsy-based biomarkers for pediatric NRSTS are limited, as most have focused on adult NRSTS patients^306,321–332^. Of note, while the molecular events driving these tumors may be the same in pediatric and adult patients, biology and prognosis often differs for reasons not yet elucidated, indicating that findings from adult patients cannot easily be translated to pediatric patients^333^. The few examples focusing on pediatric cases are briefly discussed here and indicated in Supplementary Table 8.

Athale et al.^268^ detected *EWSR1-WT1* fusion transcripts in peripheral blood (but not in bone marrow) in two out of three pediatric patients with DSRCT at diagnosis using RT-PCR. More recently, Cahn et al.^149^ used a commercially available NGS tool for detection of molecular abnormalities in plasma derived cfDNA of 45 pediatric patients including six that were suffering from NRSTS (five subtypes). They detected an *EWSR1* fusion and *PIK3CA* mutation in one DSRCT patient and no aberrations in the other five patients. Next, Colletti et al.^334^ investigated the exosomal miRNA profile in plasma samples of three adolescents suffering from DSRCT using RT-qPCR, and identified 55 miRNAs that were significantly modulated compared to healthy controls, proposing their panel as promising biomarkers for characterizing disease status in DSRCT patients. Finally, Christodoulou et al.^9^ used targeted sequencing and LP-WGS for detection of CNAs and gene fusions in plasma-derived cfDNA in 73 patients including two with SS and two with malignant peripheral nerve sheath tumor (MPNST). CNAs at diagnosis were detected in one out of two SS and one out of two MPNST patients. In addition, they identified two mutations in one out of two MPNST patients. In the other studies provided in Supplementary Table 8, either no liquid biopsy signal was detected or no discussion on NRSTS results was given due to the focus being on other pediatric tumor types^140,237,309,335,336^.

The lack of studies focused on liquid biopsies for pediatric NRSTS, together with the rarity and heterogeneity of individual NRSTS types, stresses the need for multi-center cooperative initiatives to enable prospective clinical trials with sufficiently large population cohorts. MyKIDS is an upcoming clinical trial with the aim of molecular profiling of NRSTS in children, adolescents, and young adults^337^. This trial will include a work package for investigation of ctDNA-derived biomarkers at primary diagnosis, during treatment, follow-up, and at relapse or progressive disease, using different methods (DNA methylation profiling, WES, copy number profiling, and assessment of patient specific breakpoints)^337^.

### Liver tumors

Primary pediatric liver tumors are rare, accounting for approximately 1% of all malignancies in children below 18^338^. Hepatoblastoma (HB) is the most common type (37%) followed by hepatocellular carcinoma (HCC, 21%) and very rare hepatocellular malignant neoplasm-not otherwise specified (HEM-NOS) tumors^339,340^. HB mostly affects children below the age of three, and although uncommon, incidence has been increasing recently, likely due to increased survival of premature infants^339,341^. In contrast to HB, pediatric HCC occurs mostly in children aged ≥10 ^496^. Also, etiological predisposition of pediatric HCC is different from adult HCC suggesting a possibly different pathogenesis^342^. Identification of liver tumor type is essential for treatment strategy as HB is very sensitive to chemotherapy while HCC responds poorly and management relies more on a surgical approach^338^. Treatment plans are individualized and based on patient age, histopathological and imaging information, and serum AFP levels^343^. Patients are considered cured when tumor lesions are no longer visible by imaging (including lung metastases) and/ or all (residual) tumors are completely removed surgically, and serum AFP levels have dropped back to normal^343^.

Pediatric liver cancer diagnosis and monitoring relies on serum AFP measurements, tumor biopsies, and imaging techniques^343^. However, AFP measurements can be misleading as not all liver tumors secrete AFP (more often the case for HCC)^339,344^. Also, infants (premature babies in particular) can show high physiological AFP levels^343^. In addition, discrimination among liver cancer subtypes still mostly relies on invasive tumor biopsies^345^. Finally, repeated imaging procedures can increase future oncologic risk^343^. Therefore, liquid biopsies could complement existing methods and provide a valuable tool for liver cancer diagnosis, therapy response monitoring, follow-up, and MRD detection^3^. A comprehensive overview of studies involving liquid biopsy-based biomarkers in pediatric liver cancer patients is depicted in Supplementary Table 9.

miRNAs have been described as potential liquid biopsy-based biomarkers for pediatric HB^106,129,346,347^. Firstly, Murray et al.^106^ demonstrated suitability of miR-122-5p, miR-483-30, and miR-205-5p for differentiating HB diagnosis from NB (including non-HB liver involved tumors). Next, Liu et al.^346^ suggested miR-21 as a diagnostic and prognostic biomarker in HB patients. They also showed superiority of miR-21 *versus* serum AFP levels for HB diagnosis and indicated miRNA-21 as an independent predictor of EFS for HB patients^346^. Finally, Jiao et al.^347^ suggested miRNA-34a, miRNA-34b, and miRNA-34c as diagnostic and prognostic biomarkers for HB. Although this miRNA panel was not superior *versus* serum AFP for HB diagnosis, it did show superior prognosis prediction *versus* other risk factors (AFP levels, metastasis, vascular invasion, and advanced disease stages)^347^.

Next to miRNAs, other liquid biopsies-based biomarkers have been described for implementation in pediatric liver tumors (Supplementary Table 9). Firstly, protein-based biomarkers can be used for identification of tumor-associated markers and therapeutic targets^348^. Zhao et al.^349^ used SELDI-TOF-MS as a proteomic screening tool for non-inflammatory protein markers for pediatric HB. They revealed significantly reduced expression of apolipoprotein A-1 (Apo A-I) in HB patients *versus* healthy controls, suggesting it as a biomarker for early HB diagnosis^349^. Additionally, cfDNA could provide specific genetic biomarkers for pediatric liver cancer^4,350^. Despite the low mutational burden of HB (as in most pediatric cancer types), roughly 50-90% of HB patients harbors recurrent mutations in the *CTNNB1* gene^341^. *CTNNB1* detection in with plasma-derived ctDNA from patients with localized HB has been demonstrated by Kurihara et al.^235^ and Kahana-Edwin et al.^350^ using NGS and ddPCR approaches. By serial sampling along the course of therapy and follow-up, *CTNNB1* could provide a sensitive mechanism for monitoring tumor dynamics and treatment response^350^. Finally, CTC sampling from liquid biopsies provides live information regarding disseminated tumor cell heterogeneity and response to therapy^351^. Recently, Espinoza et al.^343^ (article in preprint) demonstrated a CTC quantification method for pediatric patients suffering from HB and HCC (including the fibrolamellar carcinoma subtype) based on indocyanine green (ICG) staining followed by fluorescence microscopy and flow cytometry. ICG accumulation was specific for HB and HCC cells *versus* controls (non-malignant liver cells, non-liver tumor cells, and non-malignant cells). Also, CTC burden mirrored patients’ responses to therapy thus providing potential method for outcome prediction^343^.

Taken together, the studies discussed provide different potential liquid biopsy-based biomarkers for implementation in pediatric liver cancer oncology, including miRNAs, a protein, ctDNA, and CTCs (Supplementary Table 9). However, patient cohorts in most studies were very low, and there is no overlap in target identification among studies (except for *CTNNB1* mutations). Hence, there is an initial need for more screening studies focusing on biomarker identification^8^. Next, all studies (except for^343^) only included HB patients. This makes sense given HB is the most frequent type of pediatric liver cancer^339^. Nonetheless, given the different treatment regimens for HB and HCC, accurate diagnosis between both types and hence inclusion of HCC patients in future studies is clinically important^338^. The morphological overlap between HB and HCC can make current histological diagnosis difficult^338^. Therefore, it is desired that specific liquid biopsy-based biomarkers could complement current methods for distinction among both malignancies^3^.

## Summary of liquid biopsy-based biomarkers

Within the pediatric solid tumor type selection of this review, most literature has been published on NB. There, most studies were based on detection of characteristic genetic aberrations in plasma-derived ctDNA, and targets with high potential for clinical implementation were identified (Supplementary Table 2). Pediatric renal tumors display genetic heterogeneity and hence most studies utilized NGS approaches for biomarker identification in WT based on recurrent CNAs and mutations (Supplementary Table 3). Due to renal tumor localization, urine provides an additional interesting liquid biopsy matrix, but current methods lack sensitivity for proper implementation. miRNAs of the 371-373, 302, 367 clusters appear overexpressed in all MGCTs, with the exception of teratoma, and hence provide interesting targets (Supplementary Table 4). Nonetheless, validation in prepubertal type I GCTs is required. Osteosarcoma is genetically heterogeneous, and several liquid biopsy-based analytes have been investigated, including complex SVs, miRNAs, lncRNAs, and CTCs (Supplementary Table 5). Still, there is very little overlap in consistent targets among studies. EWS and ARMS are characterized by gene fusions that provide relevant targets for detection (*EWSR1-FLI1* and *PAX3/7-FOXO1*, respectively), while ERMS does not display such fusions and characteristic biomarkers including patient specific SVs are less established (Supplementary Tables 6 and 7 for EWS and (A/E)RMS, respectively). Few pediatric studies focused on NRSTS and liver cancer patients, and more exploratory studies are needed for proper target identification (Supplementary Tables 8 and 9, respectively). Characteristic fusions for NRSTS (such as *SS18-SSX* for SS and *EWSR1-WT1* for DSRCT), and *CTNNB1* mutations for HB provide potential starting points.

## Clinical applications and future directions

Based on published studies and future trials, clinical implementation of liquid biopsies in pediatric solid tumors is anticipated for several applications. Firstly, in a potential cancer screening setting for early diagnosis. However, this seems dubious for the general pediatric population and will likely only be relevant for specific risk groups, such as children with genetic predisposition. More relevant is risk-stratification following diagnosis and subsequent guidance of treatment management, which has already demonstrated clinical benefit in adults^352,353^. For many pediatric cancers, outcome differs significantly among risk groups, and several risk factors detectable in liquid biopsies have been discussed in our review^354^. Hence, in the context of personalized medicine and minimizing treatment-related morbidity, liquid biopsy-informed risk-stratification could guide appropriate treatment intensification or de-escalation. Serial sampling during treatment periods could similarly aid such treatment decisions. In pediatric acute lymphoblastic leukemia patients, chemotherapy was reduced successfully without affecting survival based on bone marrow-informed MRD detection throughout chemotherapy courses in a clinical setting^355,356^. Similarly, intensified therapy significantly improved outcome for patients with intermediate and high MRD levels. We discussed several pediatric studies for different solid tumor types that demonstrated significantly reductions in liquid biopsy-based biomarker signals during the course of therapy. This urges for implementation in clinical studies for informing patient-specific treatment plans as well. Next, by mitigating tumor heterogeneity, liquid biopsy sampling allows molecular profiling, monitoring of clonal evolution, and hence identification of therapy-resistant subclones, as demonstrated in adult and pediatric studies^357,358^. Deep sequencing approaches on ctDNA material could allow spatiotemporal genomics and gene expression profiling for identification of relevant cancer-associated genetic targets and pathways, and enable opportunities for targeted treatment^3^. Indeed, several liquid biopsy-based biomarkers mentioned in this review are eligible for targeted treatment, including *ALK* mutations for NB and RMS, and *EWSR1-FLI1* fusions for EWS^359^. Finally, liquid biopsy serial sampling during follow-up could monitor MRD levels, predict outcome, and inform decisions regarding need and frequency for adjuvant therapy. Relapse is a prognostic factor for poor outcome and is the primary cause for cancer related death in children^4,354^. In different adults cancers, ctDNA and/or cfDNA detection during follow-up has already been shown to outperform imaging and standard biomarkers for disease recurrence and relapse detection, with subsequent adjuvant therapy leading to improved outcome^360–364^. While we discussed some examples indicating liquid biopsy suitability for MRD monitoring versus conventional methods during follow-up, dedicated studies are lacking^237^. The upcoming MONALISA trial mentioned earlier aims to investigate liquid biopsy efficacy during follow-up versus standard methods in a clinical setting for NB patients.

In practice, during the screening and diagnosis phase, NGS approaches could complement standard of care for diagnostic molecular profiling of tumor material. There, sequencing strategies would involve targeted deep approaches in pediatric tumors with recurrent genetic or epigenetic aberrations (e.g., NB, EWS, ARMS, NRSTS, and HB) or more shallow but wider approaches for tumors with genetic heterogeneity (e.g., renal tumors, osteosarcoma, and ERMS), focusing on targets as displayed in Table 1. This genetic profiling should inform risk-stratification and subsequent treatment selection, and guide development of cost-effective and highly sensitive patient-specific ddPCR assays for the treatment and follow-up phases. During treatment, serial sampling should inform treatment efficacy and hence guide subsequent intensification or de-escalation, and monitoring of clonal evolution and therapy resistance. If the latter is observed, treatment should be changed accordingly. During follow-up, surveillance using these ddPCR assays as well as CTC detection methods can be used for MRD screening. If detected, broad NGS approaches should be utilized again for new diagnostic molecular profiling of (potentially treatment resistant) clones, before new treatment is initiated. Still, design of patient-specific ddPCR assays might not always be possible (e.g., due to absence of an appropriate chromosomal region, presence of the sequence in the normal genome, or little ctDNA (being more relevant for sarcomas)) or alternative analytes to ctDNA might be more informative^4,139,141,146,140^. Important examples include miRNA clusters (for MGCTs and osteosarcoma), CTC analysis including derived mRNA material (for NB, osteosarcoma, EWS, and RMS), and lncRNAs (for osteosarcoma) (Supplementary Tables 2–9). In such cases, assessment of preferably single and otherwise panels of simple and routinely applicable, specific, sensitive, robust, accurate, and cost-effective biomarkers using relevant platforms (e.g., CTC identification and analysis techniques, RNA seq, and miRNA profiling), depending on tumor type, will then provide most value for implementation in pediatric oncology^137,365^.

Finally, liquid biopsies could aid in identification of clonal hematopoiesis (CH). CH involves accumulation of somatic mutations in hematopoietic stem cells leading to clonal expansions of genetically distinct blood cell subpopulations without evidence of dysplasia, neoplasm, or cytopenia^8,366^. Although initially considered an age-related phenomenon, a recent study by Hagiwara et al.^367^ found significantly higher (15% vs 8.5%) mutations in CH-related genes *versus* community controls in 2860 long-term survivors of pediatric cancer with a mean follow-up time of 23.5 years. CH in survivors is associated with exposures to hazards (alkalyting agents, radiation, and bleomycin), and is now recognized as a risk for developing cardiovascular disease and hematological malignancies^367,368^. This urges the need for monitoring to investigate long-term effects of therapy-induced CH in pediatric cancer survivors. CH detection using liquid biopsies has already been suggested for different adult cancers^369–372^. In children, however, VAFs of CH remain very low and currently undetectable in many liquid biopsy assays^8^. Future efforts exploring this should be wary to avoid mix-up of signals derived from tumors and from CH, for example by targeted sequencing of paired cfDNA and white blood cells for correct identification of corresponding genetic aberrations^373^.

Taken together, methods and targets of choice inherently depend on tumor type and the clinical question at hand, most commonly being either diagnostic or prognostic. Importantly, regardless of the application or tumor type, liquid biopsy validation studies will ultimately need to demonstrate improved outcome and/ or quality of life versus standard methods in order to serve purpose in clinical oncology.

## Challenges and requirements for clinical implementation

Liquid biopsies are increasingly being investigated as non-invasive clinical biomarkers in pediatric solid tumors (Fig. 2a, b). Still, in contrast to adult oncology, liquid biopsies have not yet been implemented in daily clinical practice for several reasons. Firstly, clinical implementation requires biomarker validation in large-scale prospective trials with comparison to the standard of care at predefined timepoints^4^. Considering sampling volume restrictions and given that many pediatric solid tumor subtypes are rare, pediatric liquid biopsy material is precious, and availability is limited^8,31,47,125^. This complicates validation of findings in independent cohorts. In order to overcome these limitations, there is a need for multi-institutional collaboration with defined targets and methods in clinical trials, frequent sampling, and open access to published data^31,125,135^. Such trials are starting to emerge and examples were presented in the main text, per tumor type.

Next, biomarker validation in prospective clinical trials requires standardization and harmonization of pre-analytical conditions and protocols^374^. Pre-analytical parameters of a liquid biopsy workflow involve sampling, processing (e.g., analyte isolation or purification), and storage. For ctDNA, for example, yield, stability, and contamination with genomic DNA are affected by collection tube choice (e.g., serum *versus* plasma collection) and agitation, centrifugation force and speed, time between sampling and storage, storage temperature, freeze-thaw cycles, isolation methods, and quantification^374^. Standardized sampling conditions within and among international trials should decrease variation. Fortunately, recommendations on these conditions are emerging in recent studies^24,374,375^.

Clinical implementation of liquid biopsies also requires consistent, specific, sensitive, quick, and cost-effective methods for biomarker detection and analysis, including interpretation. Generally, relevant methods include single target approaches (e.g., PCR-based), panel approaches (targeted sequencing and arrays) and broad strategies (comprehensive sequencing)^8,40,154^. Naturally, methods of preference depend on the tumor type being investigated, involved biomarkers, and which information is required at that time. For ctDNA detection and analysis, ddPCR and NGS-based approaches are currently the most commonly used methods. ddPCR is sensitive, relatively cheap, quick, and suitable for detecting recurrent genetic aberrations such as mutations and SVs (CNAs, fusions, and translocations) with variant allele fractions (VAFs) as low as 0.001%^8,125,146,374^. Still, patients often display specific DNA breakpoints in the case of SVs, therefore requiring development of a unique assay per patient with existing knowledge of the respective genetic aberrations^4,125^. In contrast, NGS approaches allow genomic analysis without previous knowledge of tumor genetics, and can be used for investigation of individual genes, selected regions, or the entire genome^40,374^. NGS, however, is costly and hence requires balancing among sequencing breadth (proportion of genome being sequenced) and depth (number of times a nucleotide is read during sequencing). Shallow WGS approaches, for example, can detect CNAs in samples with a high fraction of ctDNA but lack sensitivity for the detection of SNVs, INDELs, and translocations. Conversely, while deep sequencing panels can identify such aberrations, they are generally restricted to targeted regions^8,125,146,376^. Deep WGS is currently still too cost-prohibitive for routinary practice^125^. Fortunately, costs are still decreasing making this method potentially feasible in the future.

ctDNA-based biomarker detection raises several challenges. Firstly, depending on cancer type, disease staging, and inherently ctDNA secretion, target scarcity among the pool of normal cfDNA fragments within peripheral blood may mask a positive signal despite ctDNA being present in the patient^140,141,377^. In pediatrics, this issue is compounded by blood volume restrictions. The maximum amount of blood that can be drawn per moment depends on the child’s age, weight, and medical condition, but is typically 0.8–0.9 mL/ kg of body weight (corresponding to 1% of total blood volume) as recommended by the European Union (most often less is taken)^378^. Also, an assay’s LOD is constrained by the number of unique molecules being present in the sample. This is primarily a concern when using sequencing assays for ctDNA detection. Sequencing to a depth greater than unique genome equivalents in the sample results in duplicate reads without improvement of the actual LOD^8^. Current efforts to mitigate these challenges include improved method sensitivity, enrichment protocols promoting yield and stability, application of sequencing error suppression algorithms, identification and removal of mutations from CH, and deep sequencing efforts with implementation of unique molecular identifiers (UMIs)^4,377,379^. UMIs tag original DNA molecules as distinct identities and therefore allow quantification of thoroughly unique molecules present in the sample^379^. Hence, these UMIs improve characterization of rare genetic alterations, reduce background signal, and maintain high positive predictive value and sensitivity^379^. Finally, several different units of measurement for ctDNA are currently used, such as ng/mL, haploid genome equivalents (copies)/ mL, fraction of total cfDNA (VAF), and copies/ mL^4^. Harmonization and guideline development will allow for better interpretation and comparisons of ctDNA analyses. Importantly, the failure rate of ctDNA-based biomarker detection and causes (e.g., too low input ctDNA, ctDNA being too fragmented, or VAF below method LOD) are currently not monitored well in pediatric studies investigating liquid biopsies. Hence, future studies should track this to elucidate their impact and clinical relevance.

## Final remarks

In this review, we provide a comprehensive overview of the literature on liquid biopsies in pediatric solid tumors and elaborate on recent developments. Especially ctDNA, but also CTCs (including derived mRNA material) and miRNAs, are the most commonly investigated liquid biopsy analytes, and could serve valuable purposes including cancer diagnosis, risk-stratification, prognostication, monitoring of therapy response and clonal evolution, MRD detection, and premature detection of relapse, as indicated by many studies (Table 1, Supplementary Tables 2–9). Taken together, liquid biopsy-derived biomarkers hold tremendous potential for application in pediatric solid tumors with the field emerging rapidly, especially during the last decade (Fig. 2a, b). Still, validation in large-scale prospective international trials with defined biomarker targets, frequent sampling, standardization of pre-analytical conditions, and defined analytical techniques is required before liquid biopsies can be implemented in the routine care for pediatric oncology patients.

## Supplementary information

Supplementary material

## Data Availability

All data generated and analyzed during this study are included in this published article (and its supplementary information files).
