# Author Correction: A comprehensive overview of liquid biopsy applications in pediatric solid tumors

**DOI:** 10.1038/s41698-024-00677-9

**Published:** 2024-09-25

**Authors:** Ferdinand W. Janssen, Nathalie S. M. Lak, Claudia Y. Janda, Lennart A. Kester, Michael T. Meister, Johannes H. M. Merks, Marry M. van den Heuvel-Eibrink, Max M. van Noesel, Jozsef Zsiros, Godelieve A. M. Tytgat, Leendert H. J. Looijenga

**Affiliations:** 1 Princess Máxima Center, Utrecht, The Netherlands; 2 Oncode Institute, Utrecht, The Netherlands; 3 Division of Imaging and Oncology, University Medical Center Utrecht, University of Utrecht, Utrecht, The Netherlands; 4 Wilhelmina Children’s Hospital-Division of CHILDHEALTH, University Medical Center Utrech, University of Utrecht, Utrecht, The Netherlands; 5 Department of Genetics, University Medical Center Utrecht, University of Utrecht, Utrecht, The Netherlands; 6 Department of Pathology, University Medical Center Utrecht, University of Utrecht, Utrecht, The Netherlands

**Keywords:** Diagnostic markers, Predictive markers, Prognostic markers, Paediatric cancer, Paediatric cancer

**Correction to:** *npj Precision Oncology* 10.1038/s41698-024-00657-z, published online 03 August 2024

In the original version of this article, an incorrect Supplementary material references file was provided. The original article has been corrected.

