# Supplementary material for: A comprehensive overview of liquid biopsy applications in pediatric solid tumors
Source: NPJ Precis Oncol. 2024 Aug 3;8:172. doi: 10.1038/s41698-024-00657-z (PMC11297996; doi:10.1038/s41698-024-00657-z)
Supplement: Supplementary file 1 — Supplementary material [file 41698_2024_657_MOESM1_ESM.pdf]

Supplementary Table no. 1: method for comprehensive literature collection on liquid biopsy investigation within pediatric solid tumors.

Step 1	Studies were collected through an advanced search on pubmed using two search strategies (below). Articles from the year 1989 up and until December 2023 were considered.	
	**Advanced Pubmed search A**	
	**Search parameter**	**Search words**
	titel/abstract	Neuroblastoma* OR wilms* OR nephoblastoma* OR malignant rhabdoid* OR renal cell carcinoma* OR renal medullary carcinoma* OR clear cell carcinoma of the kidney* OR congenital mesoblatic nephroma* OR germ cell tumor* OR teratoma* OR germinoma* OR dysgerminoma* OR embryonal carcinoma* yolk sac* OR seminoma* OR non-seminoma* OR choriocarcinoma* OR osteosarcoma* OR rhabdomyosarcoma* synovial sarcoma* OR malignant peripheral nerve sheath tumo* OR peripheral nerve sheath tumo* OR MPNST* OR PNST* OR Desmoplastic Small Round Cell Tumo* OR DSRCT* OR (Malignant rhabdoid tumo* AND soft tissue*) OR MRT* OR Alveolar Soft Part Sarcoma* OR ASPS* OR Clear Cell Sarcoma of the soft tissue* OR CCS* OR Epithelioid sarcoma* OR Ewing sarcoma* OR hepatoblastoma* OR hepatocellular carcinoma*
	titel/abstract	cancer* OR tumor* OR tumour*
	titel/abstract	blood* OR serum* OR plasma* OR urin* OR cerebrospinal fluid* OR bone marrow* OR CSF* OR saliva* OR sputum* OR ascite* OR pleural effus* OR fluid*
	titel/abstract	circulating tumor DNA* OR ctDNA* OR cell free DNA* OR cell-free DNA* OR cfDNA OR microRNA* OR miRNA* OR mRNA* OR circulating tumor cell* OR circulating tumour cell* OR CTC* OR vesicle* protein* OR platelet* OR fibroblast*
	titel/abstract	marker* OR liquid biops*
	all fields	pediatric* OR paediatric* OR prepubertal* OR child* OR adolescent*
	**Advanced Pubmed search B**	
	**Search parameter**	**Search words**
	titel/abstract	Neuroblastoma* OR wilms* OR nephoblastom* OR malignant rhabdoid* OR renal cell carcinoma* OR renal medullary carcinoma* OR clear cell carcinoma of the kidney* OR congenital mesoblatic nephroma* OR germ cell tumor* OR teratoma* OR germinoma* OR dysgerminoma* OR embryonal carcinoma* yolk sac* OR seminoma* OR non-seminoma* OR choriocarcinoma* OR osteosarcoma* OR rhabdomyosarcoma* synovial sarcoma* OR malignant peripheral nerve sheath tumo* OR peripheral nerve sheath tumo* OR MPNST* OR PNST* OR Desmoplastic Small Round Cell Tumo* OR DSRCT* OR (Malignant rhabdoid tumo* AND soft tissue*) OR MRT* OR Alveolar Soft Part Sarcoma* OR ASPS* OR Clear Cell Sarcoma of the soft tissue* OR CCS* OR Epithelioid sarcoma* OR Ewing sarcoma* OR hepatoblastoma* OR hepatocellular carcinoma*
	all fields	liquid biops*
Step 2	Review articles were collected using similar strategies, with the following exceptions: using 'all fields' instead of 'title/abstract' for all search parameters in search A, and including the (pediatric* OR paediatric* OR prepubertal* OR child* OR adolescent*) search words (all fields) in search B.	
Step 3	Titles and abstracts of identified articles (studies and reviews) were checked. Articles that focussed on liquid biopsies in one or more pediatric solid tumors were included in our initial collection. The most common exlusion criteria are indicated below.	
	Common exclusion criteria:	Articles did not focus on liquid biopsies.
		Articles were not in english.
		Articles did not focus on pediatric tumor types.
		Articles focussed on haematological or brain tumors.
Step 4	Review articles passing the previous criteria (61 passed) were included in our initial collection, and checked for additional studies that did not show up during step 1. When found, these additional studies were included in our initial collection.	
Step 5	For studies focussing on pediatric tumor types that are also common in adults (renal cell carcinoma, osteosarcoma, germ cell tumors, hepatocellular carcinoma, and non-rhabdomyosarcoma soft tissue sarcoma), we checked the patient cohort and excluded articles that focussed on adults.	
Step 6a	The remaining studies (283) formed our final collection and were comprehensively analysed (identification of pediatric patient cohort and involved tumor types, investigated liquid biopsy biomarkers, detection methods, analytes, matrices, and suggested application of the respective biomarkers). The Neuroblastoma and osteosarcoma tables (S2 and S5) are the only exceptions and more generic tables are provided instead.	
Step 6b	Studies were organized in tables according to tumor type (Tables S2-S9), and included in Table 1 and Figures 2A and 2B.	
Step 6c	Reviews were included in Table 1, Figure 2A, and Table S10	

Abbreviations: PB = peripheral blood, BM = bone marrow. All other abbreviations are provided in the main text.

Supplementary Table no. 2: overview of commonly investigated ctDNA-based biomarkers in pediatric NB patients and involved studies and methods.

Biomarker in ctDNA	Methods	References
MYCN amplification	qPCR, ddPCR, WES, WGS	140,142,144,145,235,357,380-393
ALK amplification/ mutation	qPCR, ddPCR, WES, WGS	142,235,357,380,392-395
Chromosome copy number profiling (1p and 11q loss, 17q gain)	Arrays (oncoscan, CGH, SNP, methylation), MLPA, microsatellite analysis, WGS, WES, EM-seq, ddPCR	140,144,148,152,357,386,391,395-397
Targeted genomic marker panel	targeted sequencing, qPCR, ddPCR, arrays	145-147,149,357,398,399,401,402,404
Comprehensive genome analysis	WES, WGS	235,357,391,403,404
Total cfDNA levels	WGS, qPCR, Qubit assay	138-140,235,392,405-409
Targeted methylation profiling (*RASSF1A* and *DCR2*)	methylation specific PCR, qPCR, EM-seq, ddPCR, nano-hmC-Seal	145,152,406,410,411,446
Comprehensive methylome analysis	EM-seq, WGBS, cfRRBS	152,153,236
	List of studies investigating non ctDNA-derived biomarkers such as CTCs and derived material (e.g. mRNA, miRNA, nucleosones, EVs):	51-123,400

Supplementary Table no. 3: comprehensive overview of studies involving analysis of liquid biopsy-based biomarkers in pediatric renal tumor patients.

Study and reference	Renal tumor type	Biomarker	Detection methods	Analyte	Matrix	Population	Suggested application
Schmitt et al. (2012) [183]	WT	miRNA panel	RT-qPCR, array	miRNA	PB	43	Diagnosis
Charlton et al. (2014) [412]	WT	Methylated *DMR-2*	Bisulfite sequencing	cfDNA	Serum	10	Tumor response during treatment
Murray et al. (2015) [106]	WT	miR-143-3p	RT-qPCR	miRNA	Serum	7	Differential diagnosis
Ludwig et al. (2015) [182]	WT	miRNA panel	RT-qPCR	miRNA	Serum	43	Diagnosis
Kurihara et al. (2015) [235]	WT, MRTK	*WT1* and *SMRKCB1* mutations	NGS, dPCR	cfDNA	Plasma	5	Diagnosis, treatment efficacy
Klega et al. (2018) [139]	WT	ctDNA levels, CNAs	ULP-WGS	ctDNA	Plasma	8	Disease response and genomic subclassifier identification
Biderman Waberski et al. (2018) [414]	WT	*PIK3CA*	ddPCR	cfDNA	Plasma, urine	8	Diagnosis
Ueno-Yokohata et al. (2018) [413]	CCSK	*BCOR-ITD*	PCR	cfDNA	Plasma	3	Diagnosis
Treger et al. (2018) [415]	WT	ctDNA concentration, *TP53* status	ddPCR	ctDNA	Plasma, serum, urine	4	Risk stratification and surveillance
Chen et al. (2019) [416]	RCC	Somatic mutations	targeted sequencing, WES	ctDNA	Plasma	1	Mutation detection
Jiménez et al. (2019) [179]	WT, CCSK, RCC	CNAs and SNVs	WES	ctDNA	Plasma	18	Treatment guidance, disease monitoring, follow-up
Ortiz et al. (2019) [190]	MRTK, CCSK, RCC, WT	Protein panel (prohibitin)	HR-MS, ELISA	Protein	Urine	139	Early diagnosis, therapy stratification, treatment response
Miguez et al. (2020) [180]	WT	Somatic mutations	Targeted sequencing, WES	cfDNA	Plasma, urine	5	Monitoring treatment response
Rossi et al. (2020) [417]	RCC	Counts, c-MET expression	CellSearch system	CTCs, CECs	PB	2	Prognostic marker and follow-up
He et al. (2021) [181]	WT	XIST	Array, RT-qPCR	lncRNA	PB	49	Prognostic marker
van Zogchel et al. (2021) [141]	WT	ctDNA levels and hypermethylated *RASSF1A*	ddPCR	ctDNA	Plasma	13	Pan-tumor marker
van Paemel et al. (2021) [236]	WT, MRTK, CCSK	Methylation profiling	cfRRBS	cfDNA	Plasma	19	Diagnosis
van Paemel et al. (2022) [148]	WT	CNAs	sWGS	cfDNA	Plasma	19	Prognostic classification, therapeutic stratification, tumor heterogeneity
Madanat-Harjuoja et al. (2022) [178]	WT	ctDNA levels, CNAs	ULP-WGS, targeted sequencing	ctDNA	Serum, urine	50	Prognostic marker, tumor heterogeneity
Christodoulou et al. (2023) [9]	WT, CCSK, CMN	CNA detection	Targeted sequencing, LP-WGS	cfDNA	Plasma	12	Patient monitoring for diagnosis, treatment, and recurrence
Ruas et al. (2023) [140]	WT	ctDNA levels, CNAs	dPCR	cfDNA	Plasma	14	Diagnosis, monitoring disease response

Supplementary Table no. 4: comprehensive overview of studies involving analysis of liquid biopsy-based biomarkers in pediatric MGCT patients.

Study and reference	GCT type	Biomarker	Detection method	Analyte	Matrix	Population	Suggested application
Murray et al. (2011) [209]	YST	miR-371,372,373 and 302 clusters	RT-qPCR	miRNA	Serum	1	Diagnosis and disease monitoring
Murray et al. (2016) [213]	YST, MMGCT, eMGCT, iMGCT	miR–371a–3p, miR–372–3p, miR–373–3p miR–367–3p	RT-qPCR	miRNA	Serum, CSF	25	Diagnosis and disease monitoring
Murray et al. (2020) [214]	iMGCT	miR-371a-3p	RT-qPCR	miRNA	Serum, CSF	4	Diagnosis, prognostication, and patient management
Murray et al. (2021) [196]	Choriocarcinoma	miR-371a-3p, and several within the C19MC cluster	RT-qPCR	miRNA	Serum, CSF	1	Patient management
Christodoulou et al. (2023) [9]	Not specified	CNAs (1 and 12p gain)	LP-WGS and targeted sequencing	ctDNA	Plasma	10	Diagnosis and evaluating clonal evolution
Schonberger et al. (2023) [215]	iMGCT (both non-germinoma and germinoma)	miR-371a-3p, miR-372-3p, miR-367, miR-302a, miR-302d-3p	RT-qPCR	miRNA	Serum and CSF	8	iGCT discrimination, monitor therapy response, and early relapse detection
Saliyeva et al. (2023) [216]	Various types	miR-302/367 and 371-373 clusters	RT-qPCR	miRNA	Serum	20	Diagnosis and follow-up

Supplementary Table no. 5: overview of commonly investigated liquid biopy analytes in pediatric osteosarcoma patients and involved targets, methods, and studies.

Liquid biopsy analyte	Targets	Methods	References
ctDNA	Quantification, somatic alterations (CNAs, SNVs, INDELs, SVs, translocations), methylation profiling	Targeted sequencing, ULP-WGS, sWGS CAPP-seq, ddPCR, cfRRBS	9,139,140,148,149,232,234-238, 336
CTCs	Identification and quantification, classification/ characterization, epithelial cell adhesion molecule expression	RNA-ISH, flow-cytometry, microfluidics, IMC, CanPatrol CTC technology, immunofluorescence imaging, FISH, iFISH	240,241,243-245,306,335,418-424
miRNA	Expression levels	RT-qPCR, dPCR, arrays, RNA-seq	106,247,263,425-482
lncRNA	Expression levels	RT-qPCR, dPCR, arrays, RNA-seq	483-496
EVs	Cargo analysis (proteins, RNA, ligands)	Immunogold labeling, ELISA, LC-MS, immunoblots, MALDI-TOF MS, SERS, RNA seq	233,497-500

Supplementary Table no. 6: comprehensive overview of studies involving analysis of liquid biopsy-based biomarkers in pediatric EWS patients.

Study and reference		Biomarker	Detection method	Analyte	Matrix	Population	Suggested implementation
West et al. (1997) [264]		EWS-FLI1 fusion transcripts	RT-PCR	CTC derived RNA	BM and PB	28	MRD monitoring
Zoubek et al. (1998) [265]		EWS-ETS fusion transcripts	RT-PCR	CTC derived RNA	BM	35	Prognostic
Fagnou et al. (1998) [266]		EWS-FLI1/ERG fusion transcripts	RT-PCR	CTC derived RNA	BM and PB	67	Staging and diagnosis
De Alava et al. (1998) [267]		EWS-FLI1/ERG fusion transcripts	RT-PCR	CTC derived RNA	PB	28	Therapy monitoring and prediction of disease progression
Thomson et al. (1999) [501]		t(11;22) translocations	RT-PCR	CTCs	PB and BM	9	Disease clearance
Gattenloehner et al. (1999) [122]		Myogenin	RT-PCR	mRNA	BM	5	MRD detection
Athale et al. (2001) [268]		EWS-FLI1/ERG fusion transcripts	RT-PCR	CTC derived RNA	BM and PB	31	Disease staging, follow-up, and MRD detection
Schleiermacher et al. (2003) [269]		EWS-FLI1/ERG fusion transcripts	RT-PCR	CTC derived RNA	BM and PB	172	Prognostic
Yu et al. (2012) [502]		Ccf-mtDNA levels	RT-qPCR	Ccf-mtDNA	Serum	25	Diagnosis and patient management
Nie et al. (2015) [503]		miR-125b	RT-qPCR	miRNA	Serum	63	Diagnosis
Murray et al. (2015) [106]		miR-214-3/5p, miR-92b-3p	RT-qPCR	miRNA	Serum	2	Diagnosis
Krumbholz et al. (2016) [504]		*EWSR1* fusions, ctDNA levels	ddPCR	ctDNA	Plasma	20	Therapy response
Hayashi et al. (2016) [505]		*EWS-ETS* breakpoints	ddPCR	ctDNA	Plasma	3	Relapse detection
Shukla et al. (2017) [321]		*EWSR1* fusions	ddPCR	cfDNA	Plasma	17	Disease monitoring
Hayashi et al. (2017) [306]		CTC detection and quantification, EWS-FLI1 translocation, P53 mutation	CellSieve size-based low pressure microfiltration system	CTC and derived mRNA	PB	9	Disease monitoring and relapse prediction
Klega et al. (2018) [139]		*EWSR1* translocation, ctDNA levels	Targeted sequencing	ctDNA	Plasma	11	track disease response and identify genomic subclassifiers of disease
Lee et al. (2018) [506]		type 1 EWS-FLI1 translocation	RT-qPCR	CTC derived RNA	Plasma	1	Therapy response and prognosis prediction
Shulman et al. (2018) [238]		ctDNA levels	targeted sequencing, ULP-WGS	ctDNA	Plasma	94	Diagnosis, risk stratification, monitor clonal evolution
Allegretti et al. (2018) [274]		type 1 and 2 EWS-FLI1 fusion transcripts	RT-qPCR and dPCR	ctRNA	Plasma	4	Patient monitoring and management
Benini et al. (2018) [507]		EWSR1-FLI1/ETS fusion transcripts	Immunoseparation, RT-dPCR	CTCs and derived RNA	PB	18	Prognostic and predictive purposes
Schmidkonz et al. (2020) [272]		ctDNA levels	ddPCR	ctDNA	Plasma	20	Treatment response monitoring and relapse detection
Samuel et al. (2020) [275]		sEV mRNA cargo (EWS-ETS transcripts)	RT-qPCR	sEV-derived mRNA	Plasma	10	Diagnostic and prognostic
Tombolan et al. (2020) [336]		Membrane-bound EpCAM expression	EPCAM-based CellSearch platform	CTCs	PB	2	Disseminated disease assessment
Peneder et al. (2021) [335]		*EWS-ETS* fusions, fragmentation patterns, CNA profiling	WGS, ddPCR, cfRRBS	ctDNA	PB	95	Prognostic
Bodlak et al. (2021) [276]		*EWS-FLI1/ERG* fusions	ddPCR	ctDNA, ctRNA	Plasma	5	MRD detection
Krumbholz et al. (2021) [271]		*EWSR1* fusions	ddPCR	ctDNA	Plasma	102	Early risk stratification and early prediction of treatment response
Shah et al. (2021) [237]		translocations, ctDNA levels	WGS, CAPP-seq	ctDNA	Plasma	8	Diagnosis, monitoring, and early relapse detection
van Paemel et al. (2021) [236]		Methylation profiling	cfRRBS	cfDNA	Plasma	6	Diagnosis
van Paemel et al. (2022) [148]		CNA profiling	sWGS	cfDNA	Plasma	9	Complementary assay for tissue analysis
Seidel et al. (2022) [273]		*EWS-FLI1* fusion breakpoints, ctDNA levels	ddPCR and WGS	ctDNA	Plasma	6	MRD monitoring and treatment stratification
Crow et al. (2022) [263]		miRNA profiling	miRNAseq	exosome and cell derived miRNA	Plasma	8	Diagnosis and disease monitoring
Subhash et al. (2022) [123]		CTC quantification, EWS fusion transcripts	WGS, qPCR, ddPCR	CTC	BM and PB	6	Monitoring treatment response
Cahn et al. (2022) [149]		SVs and mutations	Targeted seq	cfDNA	Plasma	5	Molecular profiling
Christodoulou et al. (2023) [9]		*EWSR1* fusions, ctDNA levels	Targeted seq, LP-WGS	cfDNA	Plasma	12	Patiënt evaluation
Ruas et al. (2023) [140]		Amount, CNA profiling	dPCR	cfDNA	Plasma	4	Diagnosis, disease response monitoring
Turaga et al. (2023) [508]		UGT3A2	LC-MS, ELISA, Western blot	sEV-derived protein	Plasma	16	Diagnosis
van Zogchel et al. (2023) [146]		Patient specific breakpoints	SNP-array, RT-qPCR, ddPCR	cfDNA	Plasma	1	MRD detection
Gelineau et al. (2023) [145]		Methylome profiling	cfRRBS	cfDNA	Plasma	1	Metastatic status determination, prognostication, and monitoring of MRD

Supplementary Table no. 7: comprehensive overview of studies involving analysis of liquid biopsy-based biomarkers in pediatric RMS patients.

Study and reference	RMS type	Biomarker	Detection method	Analyte	Matrix	Population	Suggested application
Thomson et al. (1999) [501]	ARMS	t(2;13) translocations	RT-PCR	CTCs	PB and BM	3	Disease clearance
Gattenloehner et al. (1999) [122]	ARMS	AChR	RT-PCR	mRNA	BM	5	MRD detection
Athale et al. (2001) [268]	ARMS	PAX3/7-FKHR fusion transcripts	RT-PCR	CTC derived RNA	BM and PB	13	Disease staging, follow-up, and MRD detection
Michelagnoli et al. 2003 [296]	ARMS, ERMS	MyoD1, myogenin	RT-PCR	mRNA	PB and BM	20	Diagnosis and small volume disease detection
Gallego et al. (2006) [297]	ARMS, ERMS	MyoD1, AChR, PAX3/7-FKHR	RT-PCR	mRNA	PB and BM	16	MRD detection
Sartori et al. (2006) [298]	ARMS, ERMS	MyoD1, myogenin, PAX-FKHR transcripts	RT-PCR	mRNA	BM	40	MDD detection
Miyachi et al. (2010) [509]	Not specified	miR-206	RT-PCR	miRNA	Serum	10	RMS detection
Krsková et al. (2010) [299]	ARMS	PAX3/7-FKHR, MyoD1	RT-qPCR	mRNA	PB and BM	33	MDD detection
Murray et al. (2015) [106]	ERMS	miR panel	RT-qPCR	miRNA	Serum	3	Diagnosis
Hayashi et al. (2017) [306]	ARMS, ERMS	CTC amount	CellSieve, single cell RNA-seq	CTCs	PB	4	Disease response monitoring, prediction of metastatic relapse
Klega et al. (2018) [139]	ARMS	ctDNA levels	ULP-WGS	ctDNA	Plasma	7	Monitoring disease response, identification of genomic subclassifiers
Eguchi-Ishimae et al. (2019) [307]	ARMS	*PAX3-FOXO1* fusion gene	qPCR	ctDNA and CTCs	PB and BM	1	Monitoring tumor burden
Ghamloush et al. (2019) [510]	ARMS, ERMS	miR-486-5p	Microarray	Exosomal miRNA	Serum	Not specified	RMS detection
Tombolan et al. (2020) [511]	ARMS, ERMS	miR-26a	RT-qPCR, ddPCR	miRNA	Plasma	30	Diagnostic, prognostic
Poli et al. (2020) [303]	ARMS, ERMS	IGFBP2 protein and autoantibodies	Direct and indirect ELISA	circulating protein	Plasma	114	Diagnostic, prognostic
Tombolan et al. (2020) [336]	ARMS, ERMS	Membrane-bound EpCAM expression	EPCAM-based CellSearch platform	CTCs	PB	7	Disseminated disease assessment
van Zogchel et al. (2021) [141]	Not specified	Hypermethylated *RASSF1A*	ddPCR	cfDNA	Plasma	14	RMS detection
Shah et al. (2021) [237]	ARMS	Recurrent translocation breakpoints	CAPP-seq	ctDNA	Plasma	4	Diagnosis, monitoring, early relapse detection
Peneder et al. (2021) [335]	ARMS, ERMS	WGS, ddPCR, cfRRBS	*EWS-ETS* fusions, fragmentation patterns, CNA profiling	ctDNA	PB	12	prognostic
Lak et al. (2021) [289]	ARMS, ERMS	Targeted marker panel	RT-qPCR	CTC derived mRNA	PB and BM	99	Disseminated disease detection at diagnosis, conventional risk stratification
van Paemel et al. (2021) [236]	ARMS, ERMS	Methylation profiling	cfRRBS	cfDNA	Plasma	17	Diagnosis

van Paemel et al. (2022) [148]	Not specified	CNA profiling	sWGS	cfDNA	Plasma	10	Prognostic classification, therapeutic stratification, tumor heterogeneity
Tombolan et al. (2022) [308]	ARMS, ERMS	CTC and DCT amount, patient specific somatic variants	EpCAM-based CellSearch platform, WES, ddPCR	CTCs, DCTs, cfDNA	PB and BM	17	Disseminated disease assessment, longitudinal patient monitoring
Cahn et al. (2022) [149]	Not specified	SVs and mutations	Targeted seq	cfDNA	Plasma	3	Molecular profiling
Ruhen et al. (2022) [300]	ARMS, ERMS	ctDNA levels	ddPCR, targeted sequencing, WES	ctDNA	Plasma	28	Disease burden, treatment response
Urla et al. (2022) [304]	ARMS, ERMS	TKTL1, APO10	EDIM, flow-cytometry	Monocytes	PB	29	Therapeutic guidance, monitoring of recurrence
Stegmaier et al. (2022) [309]	ARMS	PAX3/7-FOXO1 fusion transcripts	RT-qPCR	Exosomal cfRNA	Plasma	65	Diagnosis, monitoring
Lak et al. (2023) [302]	ARMS, ERMS	Methylation profiling, ctDNA amount, *RASSF1A* hypermethylation, RNA marker panel	shWGS, cfRRBS, ddPCR	ctDNA	Plasma	57	Diagnosis, prognosis
Christodoulou et al. (2023) [9]	ARMS, ERMS	Patient specific genome-wide CNAs and fusion genes	lp-WGS, targeted sequencing	cfDNA	Plasma	5	Patient monitoring for diagnosis, treatment, and recurrence
Ruas et al. (2023) [140]	Not specified	cfDNA levels, CNAs	dPCR	cfDNA	Plasma	4	Monitoring disease response
van Zogchel et al. (2023) [146]	ARMS	Patient-specific markers for cfDNA detection	TLA, TLC, ddPCR	cfDNA	Plasma	2	MRD detection
Abbou et al. (2023) [301]	ARMS, ERMS	ctDNA levels, CNAs, patient-specific rearrangements and SNVs	ULP-WGS, targeted sequencing	ctDNA	Serum	124	Prognosis
Gelineau et al. (2023) [145]	ERMS	Targeted marker panel	RT-qPCR, ddPCR, cfRRBS	cfDNA	PB and BM	2	Metastatic status, prognostication, and disease response monitoring
de Traux de Wardin et al. (2023) [305]	ARMS, ERMS	Targeted marker panel (SNVs, fusion genes, CNAs)	Targeted seq, WES, WGS	ctDNA	Plasma	10	Disease response monitoring

Supplementary Table no. 8: comprehensive overview of studies involving analysis of liquid biopsy-based biomarkers in pediatric NRSTS tumors.

Reference	NRSTS subtype	Marker	Methods	Analyte	Matrix	Population	Suggested application
Athale et al. (2001) [268]	DSRCT	EWS-WT1 fusion transcript	RT-PCR	CTC derived RNA	BM and PB	3	Disease staging, follow-up, and MRD detection
Colletti et al. (2019) [334]	DSRCT	miRNA panel	RT-qPCR	miRNA	Exosomes	3	DSRCT disease status and therapeutic response
Tombolan et al. (2020) [336]	SS	Membrane-bound EpCAM expression	EPCAM-based CellSearch platform, RT-qPCR	CTCs	PB	1	N/A (no liquid biopsy signal)
Peneder et al. (2021) [335]	SS	SVs	WGS, ddPCR, cfRRBS	cfDNA	PB	3	N/A (no elaboration on results)
Shah et al. (2021) [237]	SS	Translocations (*SSX1-SS18*), ctDNA levels	WGS, CAPP-seq	ctDNA	Plasma	1	N/A (no liquid biopsy signal)
Stegmaier et al. (2022) [309]	SS	SYT-SSX fusion transcripts	RT-qPCR	Cell-free exosomal RNA	Plasma	15	N/A (no liquid biopsy signal)
Cahn et al. (2022) [149]	Five subtypes	SVs and mutations	Targeted seq	cfDNA	Plasma	6	Molecular profiling
Ruas et al. (2023) [140]	Leiomyosarcoma	cfDNA levels, CNAs	dPCR	cfDNA	Plasma	1	N/A (no elaboration on results)
Christodoulou et al. (2023) [9]	SS, MPNST	CNAs, and mutations	Targeted seq, LP-WGS	cfDNA	Plasma	4	Patient evaluation

Supplementary Table no. 9: comprehensive overview of studies involving analysis of liquid biopsy-based biomarkers in pediatric liver cancer patients.

Study and reference	Liver tumor type	Biomarker	Detection method	Analyte	Matrix	Population	Suggested application
Murray et al. (2015) [106]	HB	miR-122-5p, miR-483-3p and miR-205-5p	RT-qPCR	miRNA	Serum	4	Diagnosis
Zhao et al. (2015) [349]	HB	Apo A-I	qPCR, ELISA	Protein	Serum	30	Diagnosis
Kurihara et al. (2015) [235]	HB	*CTNNB1* and *APC* mutations	Targeted seq, dPCR	cfDNA	Plasma	6	Diagnosis, treatment efficacy
Liu et al. (2016) [346]	HB	miR-21	RT-qPCR	miRNA	Whole blood	32	Diagnosis and prognosis
Jiao et al. (2017) [347]	HB	miR-34a, miR-34b, and miR-34c	RT-qPCR	miRNA	Whole blood	89	Diagnosis and prognosis
Kahana-Edwin et al. (2020) [350]	HB	*CTNNB1*	ddPCR	ctDNA	Plasma	3	Treatment response
Christodoulou et al. (2023) [9]	HB	CNA detection	Targeted seq, LP-WGS	cfDNA	Plasma	3	Patient monitoring for diagnosis, treatment, and recurrence
Espinoza et al. (2023) [343]*	HB and HCC (including FLC subtype)	Quantification	Fluorescence microscopy and flow cytometry	CTC	Whole blood	14	Prognostication
*: at time of publication, this article is in a preprint and has not been peer-reviewed yet.							

Supplementary Table no. 10: comprehensive overview of review articles focussing on liquid biopsy-based biomarkers in pediatric cancer patients.

Study and reference	Title
Zhenjian et al. (2022) [124]	Advances in liquid biopsy in neuroblastoma
Almstrup et al. (2020) [200]	Application of miRNAs in the diagnosis and monitoring of testicular germ cell tumours.
Li et al. (2018) [512]	Application of liquid biopsy in bone and soft tissue sarcomas: Present and future.
Abbou et al. (2018) [125]	Assessment of circulating tumor DNA in pediatric solid tumors: The promise of liquid biopsies.
Murray et al. (2015) [513]	Biology of childhood germ cell tumours, focussing on the significance of microRNAs
Zheng et al. (2023) [165]	Biomarkers for patients with Wilms tumor: a review
Takami et al. (2022) [514]	Biomarkers for risk-based treatment modifications for CNS germ cell tumors: Updates on biological underpinnings, clinical trials, and future directions
Charlton et al. (2015) [164]	Biomarkers to detect Wilms tumors in pediatric patients: where are we now?
Salguero-Aranda et al. (2020) [279]	Breakthrough Technologies Reshape the Ewing Sarcoma Molecular Landscape
Raimondi et al. (2017) [248]	Circulating biomarkers in osteosarcoma: new translational tools for diagnosis and treatment
Andersson et al. (2020) [31]	Circulating cell-free tumor DNA analysis in pediatric cancers.
Moonmuang et al. (2021) [249]	Circulating Long Non-Coding RNAs as Novel Potential Biomarkers for Osteogenic Sarcoma
Gally et al. (2021) [250]	Circulating MicroRNAs as Novel Potential Diagnostic Biomarkers for Osteosarcoma: A Systematic Review
Fankhauser et al. (2022) [515]	Circulating MicroRNAs for Detection of Germ Cell Tumours: A Narrative Review
Leao et al. (2021) [202]	Circulating MicroRNAs, the Next-Generation Serum Biomarkers in Testicular Germ Cell Tumours: A Systematic Review
Bottani et al. (2019) [516]	Circulating miRNAs as Diagnostic and Prognostic Biomarkers in Common Solid Tumors: Focus on Lung, Breast, Prostate Cancers, and Osteosarcoma
Kojima et al. (2023) [126]	Circulating Tumor Cells and Tumor Progression, Metastasis, and Poor Prognosis in Patients With Neuroblastoma
Wei et al. (2020) [43]	Circulating tumor DNA in neuroblastoma
Yang et al. (2023) [42]	Circulating tumor cells in neuroblastoma: Current status and future perspectives.
Doculara et al. (2022) [4]	Circulating Tumor DNA in Pediatric Cancer.
Trigg et al. (2020) [127]	Diagnostic accuracy of circulating-free DNA for the determination of MYCN amplification status in advanced-stage neuroblastoma: a systematic review and meta-analysis
Wang et al. (2014) [251]	Diagnostic and prognostic value of circulating miR-21 for cancer: A systematic review and meta-analysis
Jezierska et al. (2022) [517]	Diagnostic, Prognostic and Predictive Markers in Pediatric Germ Cell Tumors—Past, Present and Future
Uemura et al. (2019) [128]	Dynamics of Minimal Residual Disease in Neuroblastoma Patients
Galardi et al. (2019) [129]	Exosomal MiRNAs in Pediatric Cancers.
Dean et al. (2018) [252]	From genomics to metabolomics: emerging metastatic biomarkers in osteosarcoma
Vellichiram et al. (2021) [130]	Fusion genes as biomarkers in pediatric cancers: A review of the current state and applicability in diagnostics and personalized therapy
Gholamin et al. (2018) [131]	GD2-targeted immunotherapy and potential value of circulating microRNAs in neuroblastoma
Lobo et al. (2019) [519]	Human Germ Cell Tumors are Developmental Cancers: Impact of Epigenetics on Pathobiology and Clinic
Ucci et al. (2022) [231]	Liquid biopsies in primary and secondary bone cancers
Lobo et al. (2021) [197]	Liquid Biopsies in the Clinical Management of Germ Cell Tumor Patients: State-of-the-Art and Future Directions
Sundby et al. (2022) [8]	Liquid biopsies in pediatric oncology: opportunities and obstacles.
Segura et al. (2022) [132]	Methodological advances in the discovery of novel neuroblastoma therapeutics
Sasaki et al. (2019) [520]	MicroRNA-Based Diagnosis and Treatment of Metastatic Human Osteosarcoma
Liu et al. (2017) [253]	MicroRNAs as a novel class of diagnostic biomarkers for the detection of osteosarcoma: a meta-analysis
Andreeva et al. (2023) [133]	MicroRNAs as prospective biomarkers, therapeutic targets and pharmaceuticals in neuroblastoma
Galardi et al. (2018) [134]	MicroRNAs in Neuroblastoma: Biomarkers with Therapeutic Potential.
Zhang et al. (2015) [254]	MicroRNAs in osteosarcoma
De Martino et al. (2021) [521]	miRNAs and Biomarkers in Testicular Germ Cell Tumors: An Update
Chavarriaga et al. (2023) [522]	miRNAs for testicular germ cell tumours: Contemporary indications for diagnosis, surveillance and follow-up
Zhang et al. (2018) [261]	Molecular Assessment of Circulating Exosomes towards Liquid Biopsy Diagnosis of Ewing Sarcoma Family of Tumors
Lerone et al. (2021) [50]	Molecular Genetics in Neuroblastoma Prognosis
Hettmer et al. (2022) [286]	Molecular testing of rhabdomyosarcoma in clinical trials to improve risk stratification and outcome: A consensus view from European paediatric Soft tissue sarcoma Study Group, Children's Oncology Group and Cooperative Weichteilsarkom-Studiengruppe.

Zeuschner et al. (2020) [523]	Non-coding RNAs as biomarkers in liquid biopsies with a special emphasis on extracellular vesicles in urological malignancies
Trigg et al. (2019) [47]	Opportunities and challenges of circulating biomarkers in neuroblastoma.
Aran et al. (2021) [228]	Osteosarcoma, chondrosarcoma and Ewing sarcoma: Clinical aspects, biomarker discovery and liquid biopsy
Lakpour et al. (2021) [524]	Potential biomarkers for testicular germ cell tumour: Risk assessment, diagnostic, prognostic and monitoring of recurrence
Gao et al. (2020) [255]	Potential diagnostic value of miRNAs in peripheral blood for osteosarcoma: A meta-analysis
Rizk et al. (2019) [270]	Precision medicine approaches for the management of Ewing sarcoma: current perspectives
Looijenga et al. (2019) [525]	Predicting Gonadal Germ Cell Cancer in People with Disorders of Sex Development; Insights from Developmental Biology
Weiser et al. (2019) [3]	Progress toward liquid biopsies in pediatric solid tumors
Bhavsar et al. (2022) [136]	Recent advances in the roles of exosomal microRNAs in neuroblastoma
Wang et al. (2020) [256]	Role of exosomal miR-21 in the tumor microenvironment and osteosarcoma tumorigenesis and progression (Review).
Perut et al. (2019) [518]	The Emerging Roles of Extracellular Vesicles in Osteosarcoma
van Paemel et al. (2019) [135]	The pitfalls and promise of liquid biopsies for diagnosing and treating solid tumors in children: a review
Murray et al. (2016) [526]	The present and future of serum diagnostic tests for testicular germ cell tumours
Ditonno et al. (2023) [527]	The Role of miRNA in Testicular Cancer: Current Insights and Future Perspectives
De Carvalho et al. (2016) [137]	Translating microRNAs into biomarkers: What is new for pediatric cancer?
Walz et al. (2023) [161]	Tumor biology, biomarkers, and liquid biopsy in pediatric renal tumors.
Varkey et al. (2021) [35]	Tumor-Educated Platelets: A Review of Current and Potential Applications in Solid Tumors
Shulman et al. (2020) [528]	Using Liquid Biopsy in the Treatment of Patient with OS
